# Vegetation type, not the legacy of warming, modifies the response of microbial functional genes and greenhouse gas fluxes to drought in Oro-Arctic and alpine regions

**DOI:** 10.1093/femsec/fiad145

**Published:** 2023-11-10

**Authors:** Ellen L Fry, Deborah Ashworth, Kimberley A J Allen, Nathalie Isabelle Chardon, Christian Rixen, Mats P Björkman, Robert G Björk, Thomas Stålhandske, Mathias Molau, Brady Locke-King, Isabelle Cantillon, Catriona McDonald, Hongwei Liu, Franciska T De Vries, Nick J Ostle, Brajesh K Singh, Richard D Bardgett

**Affiliations:** School of Earth and Environment Sciences, University of Manchester, Oxford Road, Manchester, M13 9PL, United Kingdom; Department of Biology, Edge Hill University, St Helens Road, Ormskirk, Lancashire, L39 4AP, United Kingdom; School of Earth and Environment Sciences, University of Manchester, Oxford Road, Manchester, M13 9PL, United Kingdom; School of Earth and Environment Sciences, University of Manchester, Oxford Road, Manchester, M13 9PL, United Kingdom; Biodiversity Research Centre, University of British Columbia, 2212 Main Mall Vancouver, BC V6T 1Z4, Canada; WSL Institute for Snow and Avalanche Research SLF, Flüelastrasse 11, CH-7260 Davos Dorf, Switzerland; Swiss Federal Institute for Forest, Snow and Landscape Research (WSL), Flüelastrasse 11, 7260 Davos Dorf, Switzerland; Climate Change, Extremes and Natural Hazards in Alpine Regions Research Centre CERC, Flüelastrasse 11, 7260 Davos Dorf, Switzerland; Department of Earth Sciences, University of Gothenburg, Box 100 405 30 Gothenburg, Gothenburg, Sweden; Gothenburg Global Biodiversity Centre, Box 100 405 30 Gothenburg, Gothenburg, Sweden; Department of Earth Sciences, University of Gothenburg, Box 100 405 30 Gothenburg, Gothenburg, Sweden; Gothenburg Global Biodiversity Centre, Box 100 405 30 Gothenburg, Gothenburg, Sweden; Department of Earth Sciences, University of Gothenburg, Box 100 405 30 Gothenburg, Gothenburg, Sweden; Department of Earth Sciences, University of Gothenburg, Box 100 405 30 Gothenburg, Gothenburg, Sweden; Department of Biology, Edge Hill University, St Helens Road, Ormskirk, Lancashire, L39 4AP, United Kingdom; Department of Biology, Edge Hill University, St Helens Road, Ormskirk, Lancashire, L39 4AP, United Kingdom; Hawkesbury Institute for the Environment, Western Sydney University, Bourke Street, Penrith, NSW, Australia; Hawkesbury Institute for the Environment, Western Sydney University, Bourke Street, Penrith, NSW, Australia; Institute for Biodiversity and Ecosystem Dynamics, University of Amsterdam, 1090 GE Amsterdam, the Netherlands; Lancaster Environment Centre, Lancaster University, Bailrigg, Lancaster, LA1 4YW, United Kingdom; Hawkesbury Institute for the Environment, Western Sydney University, Bourke Street, Penrith, NSW, Australia; Global Centre for Land-Based Innovation, Western Sydney University, Bourke Street, Penrith, NSW, Australia; School of Earth and Environment Sciences, University of Manchester, Oxford Road, Manchester, M13 9PL, United Kingdom

**Keywords:** ITEX, greenhouse gases, functional genes, carbon dioxide, methane, microbial community, resistance, resilience

## Abstract

Climate warming and summer droughts alter soil microbial activity, affecting greenhouse gas (GHG) emissions in Arctic and alpine regions. However, the long-term effects of warming, and implications for future microbial resilience, are poorly understood. Using one alpine and three Arctic soils subjected to *in situ* long-term experimental warming, we simulated drought in laboratory incubations to test how microbial functional-gene abundance affects fluxes in three GHGs: carbon dioxide, methane, and nitrous oxide. We found that responses of functional gene abundances to drought and warming are strongly associated with vegetation type and soil carbon. Our sites ranged from a wet, forb dominated, soil carbon-rich systems to a drier, soil carbon-poor alpine site. Resilience of functional gene abundances, and in turn methane and carbon dioxide fluxes, was lower in the wetter, carbon-rich systems. However, we did not detect an effect of drought or warming on nitrous oxide fluxes. All gene–GHG relationships were modified by vegetation type, with stronger effects being observed in wetter, forb-rich soils. These results suggest that impacts of warming and drought on GHG emissions are linked to a complex set of microbial gene abundances and may be habitat-specific.

## Teaser

Over Oro-Arctic and alpine regions, the legacy of warming is less important than vegetation type when determining the response of microbial functional genes to drought.

## Introduction

**Figure 1.**
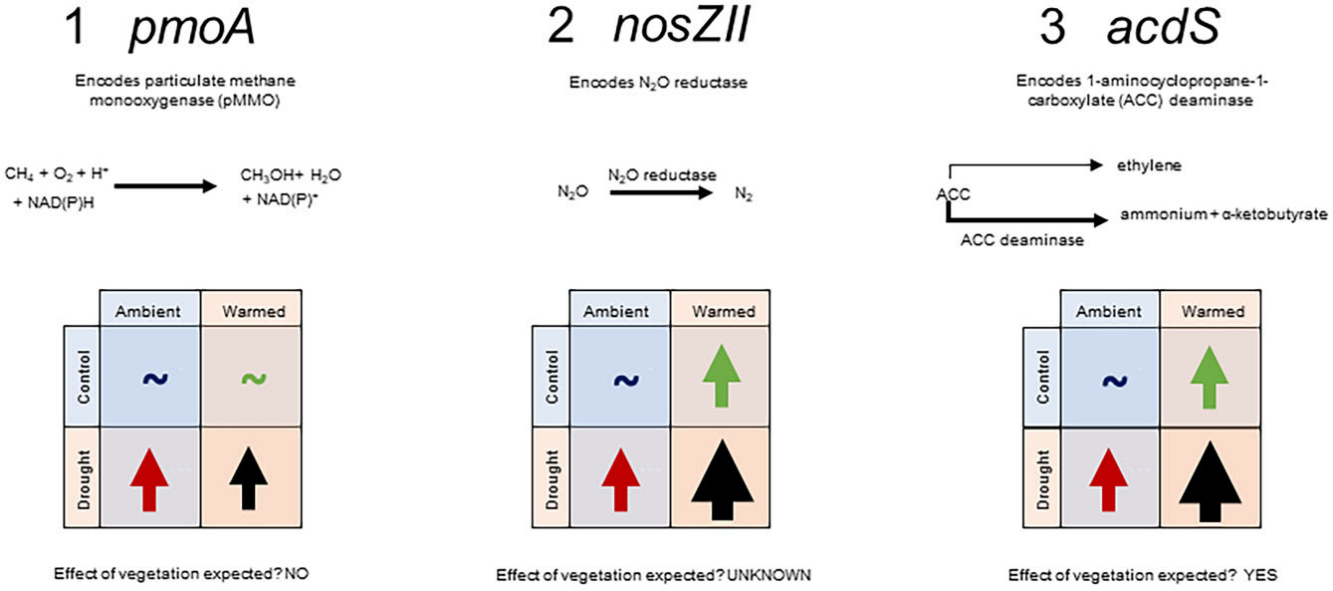
Expectations of functional gene response to long-term warming and drought. A tilde infers no expected effect. Colours to provide contrast. Stronger expected effects on gene expression are shown as larger arrows. We also include the pathway of the enzyme that the gene encodes for completeness. Further expectation of effect of vegetation type is mentioned, though not emphasized due to lack of literature. (A) *pmoA* is expected to have a low or negligible response to warming (Keuschnig et al. 2022), and an increase under drought, leading to a reduction in CH_4_ emissions (Olefeldt et al. 2013). (B) *nosZII* genes are thought to increase under drought and warming, although evidence is currently limited (Xu et al. 2020a). (C) expected effects of drought and warming on *acdS* expression. There is currently limited evidence for responses to changes in climatic conditions, but since the gene is expressed by plant-associated microbes, we anticipate a strong effect of vegetation type (Nikolic et al. 2011, Liu et al. 2019a).

High latitude and alpine environments are experiencing climate change more rapidly and severely than temperate areas (Grabherr et al. 2010, Singh et al. 2010, Palomo 2017, Rantanen et al. 2022). Combined effects of warming and changes in precipitation patterns are likely to have profound effects on soil microbial communities in these areas (Olefeldt et al. 2013), potentially resulting in large shifts in microbial community composition and functioning. With the release of physiological constraints created by low temperatures and waterlogging, there is potential for soil microbial activity to increase in response to simultaneous warming and drought (Sheik et al. 2011, Seo et al. 2015, Commane et al. 2017). As Arctic and alpine tundra are globally important carbon (C) stores, any increase in microbial activity could result in significant increases in greenhouse gas (GHG) emissions, further accelerating climate change (Seo et al. 2015). Additionally, warming will result in shortened snow cover duration and increased permafrost melt, and encroachment of trees and altered vegetation (Hagedorn et al. 2019), which has been shown to enhance soil C loss due to increased microbial decomposition (Hartley et al. 2012, García Criado et al. 2020). GHGs are generated through microbial transformation of soil C and nitrogen (N) pools, which leads to the emission of carbon dioxide (CO_2_), methane (CH_4_), and nitrous oxide (N_2_O). Various guilds of microbes have roles in the emissions of these gases, and in converting them to less potent gases in terms of global warming potential (Fig. 1; Martins et al. 2017, Lafuente et al. 2020). We can identify specific genes that directly code for enzymes which degrade or synthesize GHGs. However, while we have a relatively good understanding of the role of microbial functional genes involved in GHG emissions, little is known about how they respond to warming and drought either individually or in combination, or how these responses regulate GHG emissions (Li et al. 2020, Zhang et al. 2022). The identity of these gases is also critical as they have different global warming effects: CO_2_ has a weak effect on climate change but is very persistent in the atmosphere, while CH_4_ and N_2_O are short-lived, they are more potent (Shine et al. 2005). As such, the abundance of functional genes that code for GHG reducing enzymes could be critical to assessing future impacts of individual gases.

While CO_2_ fluxes are likely to be governed by a complex mixture of plant and microbial activity, CH_4_ emissions are largely microbially mediated: methanogenic microbes undertake methanogenesis using organic sugars as a substrate, while methanotrophs break the extremely strong C–H bond in the CH_4_ via expression of functional genes, predominantly *pmoA* (although other genes play important roles including *mmoX* and *mxaF*), under aerobic conditions (Singh et al. 2010). The *pmoA* genes encode the enzyme particulate CH_4_ monooxygenase, which catalyzes the conversion of CH_4_ to methanol (Liu et al. 2015). While warming might increase the emissions of CH_4_ from soils, the temperature sensitivity of the processes underpinning methanogenesis and methanotrophy is lower in tundra soils than in temperate soils (Tveit et al. 2015). Therefore, at low temperatures, CH_4_ emissions are high, but with warming, the accompanying increase in CH_4_ in tundra is smaller compared with temperate soils, which could be due to increased expression of *pmoA* and consequent consumption of CH_4_ (Fig. 1A; Tveit et al. 2015). There is evidence from paddy fields, under extremely cold and waterlogged soils, that CH_4_ emissions are strongly dependent on soil organic matter content, while in drier, warmer soils the CH_4_ emissions are linked to the abundance of *pmoA* (Xu et al. 2020b). However, the bulk of research on functional gene responses to climate changes has been carried out on highly organic soils, and little is known about more mineral-based soils (Keuschnig et al. 2022), which also dominates many tundra and alpine regions (Walker et al. 2005).

The denitrifying genes *nosZI* and *nosZII* have a parallel role to *pmoA*, producing gene products which convert N_2_O to N_2_ gas, and thus decreasing the global warming potential of the soil (Orellana et al. 2014). Much work has been carried out looking at *nosZ* genes in wetlands, showing that functional genes associated with N processing tend to increase with warming (Zhang et al. 2015, Wang et al. 2018). While *nosZI* is sensitive to soil moisture, there is no evidence of an impact of warming. For *nosZII*, the only clear effect is a negative relationship with N_2_O emissions, and unclear effects of drought and warming (Xu et al. 2020a). Overall, there is little information about an interaction between warming and drought for both *pmoA* and *nosZ* genes, and while climate change potentially could mean increases in water from snowmelt and permafrost thaw, IPCC projections also indicate regional variation in high latitudes (IPCC 2023). There could equally be an increase in the frequency and amplitude of summer or late season drought, resulting in a drop in the water table (Li et al. 2019), and/or an increase in evapotranspiration due to higher plant productivity (Keuschnig et al. 2022) leading to drier soils. The consequences for functional gene abundances and the link with GHG emissions are therefore unclear, as is the potential for recovery from extended drought.

The impact of warming and drought on GHG fluxes is also likely influenced by vegetation, because the varying quality and quantity of litter inputs and root exudates dictate microbial substrate availability. Further, vegetation types are likely to influence soil abiotic properties such as porosity and aggregate stability, which will in turn affect diffusion rates of gases, soil moisture conditions, and microclimate (Szymański 2017). Another important question is whether microbial stress genes have the potential to reduce GHG fluxes through encoding enzymes that reduce plant stress. The 1-aminocyclopropane-1-carboxylate (ACC) deaminase gene *acdS* is released by plant-associated rhizobacteria and bacterial endophytes and is known to aid stress relief in plants (Nikolic et al. 2011, Liu et al. 2019a). The mechanism for relieving stress in plants is by breaking down ACC, a precursor of ethylene, to ammonium and α-ketobutyrate (Gupta and Pandey 2019; Fig. 1). To our knowledge, no studies to date have considered the abundance of *acdS* in response to warming, with the majority looking at stresses such as salinity (Orhan et al. 2016, Liu et al. 2019a) or heavy metals (Loganathan et al. 2015). However, it is likely that both the plants and rhizobacteria will increase activity in response to warming and drought, and that this will mean increased expression of *acdS*.

The overarching aim of this study was to test whether long-term experimental warming modifies the functional response of microbial communities to drought and determine the consequences for GHG emissions. We tested this with four soil samples taken from two established International Tundra EXperiment sites (ITEX; Henry and Molau 1997, Henry et al. 2022) subject to long term experimental warming: a high latitude Oro-Arctic site in Sweden, Latnjajaure Field Station (three vegetation types with matched pairs of warmed and ambient plots), and an alpine site in Val Bercla, Switzerland (one vegetation type with warmed and ambient plots). Soils taken from experimental plots were subject to an experimental drought in the laboratory, and the impact of the drought on functional gene abundance and GHG flux immediately after the drought period (i.e. resistance; note that an increase relative to the control also constitutes low resistance) and its recovery back towards the control values (i.e. resilience) was assessed over a period of 56 days after rewetting. First, we hypothesized that due to release of the cold and wet constraints on microbes, long-term warming will increase functional gene abundances involved in GHG emissions, but that this might further increase gene abundance in response to drought, reducing their resistance. However, we expect that this legacy of warming will increase the resilience of the functional genes, resulting in more rapid recovery of gene abundance after drought in warmed soils compared to ambient conditions (Xu et al. 2020b). Second, we expected that GHG emissions will increase in the warming legacy plots relative to ambient plots due to increases in kinetic energy, despite concomitant increases in GHG mitigating genes. Furthermore, the combination of drought and warming will increase GHG emissions, as the conditions become dryer and have higher oxygen availability. Third, we hypothesized that the effect of long-term warming and subsequent drought on functional gene abundances and GHG fluxes are moderated by vegetation type, with effects being stronger for soils of wetter, more forb or graminoid-rich plant communities than drier, shrub-dominated communities.

## Methods

### Experimental set up

The long-term warming design used in this study consisted of open top chambers (OTCs; Hollister et al. 2022), which had a basal area of 1 m^2^ and passively warmed the interior air and soil (average warming 1–3°C; Marion et al. 1997). We used soils from an Oro-Arctic environment (Latnjajaure field station), and an alpine environment (Val Bercla field station), with four soil types that represent a gradient of both soil moisture and soil C and N, as well as contrasting vegetation types (Table 1). At Latnjajaure field station, Sweden (68°22N 18°13E, 981 m a.s.l, average January and July temperature −8.9°C and 8.6°C, respectively, mean precipitation 846 mm, ranging between 600 and 1100mm yr^−1^) the year-round experiment was set up in 1993–1994 across diverse vegetation types dominated by different plant species. The experiment consisted of a full factorial design, with warming as one treatment and vegetation type as another. Each treatment was replicated five times per plant community (Scharn et al. 2022). Here, we collected soils from three vegetation types (three levels of the vegetation treatment), forming a moisture gradient, where the phenology, germination and growth of the dominant plant species have been shown to be highly responsive to the warming treatment (Scharn et al. 2021, 2022), namely: a wet meadow environment characterized by *Bistorta vivipara, Carex bigelowii, Calamagrostis stricta*, and *Poa arctica*, a dry heath environment characterized by *Betula nana, Cassiope tetragona, Salix herbacea*, and *Empetrum nigrum*, and a tussock tundra site, characterized by *Eriophorum vaginatum, Phyllodoce caerulea, Vaccinium vitis-idaea*, and *S. herbacea* (Henry and Molau 1997, Molau 1997, Scharn et al. 2021, 2022). In total, 30 soil samples were obtained at Latnjajaure, including soils taken from warmed and ambient plots and three vegetation types, each replicated five times (Table 1).

**Table 1.** Soil properties at the start of the laboratory incubations. pH is taken from Scharn et al. (2021) for the Latnjajaure samples.

		pH		Total C (%)		Total N (%)	
Field station	Vegetation type	Ambient	Warmed	Ambient	Warmed	Ambient	Warmed
Latnjajaure	Wet meadow	6.10(0.15)	6.00(0.18)	15.13(4.50)	12.98(4.12)	1.04(0.31)	0.82(0.24)
Latnjajaure	Dry heath	4.91(0.11)	4.72(0.08)	12.32(3.62)	11.45(2.54)	0.58(0.19)	0.51(0.10)
Latnjajaure	Tussock tundra	5.11(0.10)	5.02(0.08)	3.44(0.77)	2.28(0.26)	0.16(0.04)	0.11(0.01)
Val Bercla	Alpine			1.23(0.16)	0.89(0.16)	0.11(0.01)	0.09(0.01)

At the alpine site Val Bercla, Switzerland (46°29N, 9°35E, 2490 m a.s.l, 20% NNW facing slope, average January and July temperature −6.4°C and 9.7°C, respectively, mean precipitation 1605 mm), the experiment was set up in 1994 on loamy sand (Stenström et al. 1997). At this site, the vegetation was more homogeneous and dominated by the forb *Saxifraga oppositifolia*, so it comprises one level of the vegetation treatment. *Saxifraga oppositifolia* has been shown to be responsive to warming at the northerly edge of its range (Wookey et al. 1993). A total of 18 soil samples were collected from warmed and ambient plots (average OTC warming 1.0°C), with each treatment being replicated nine times.

Soils to 10 cm deep were collected in July 2018 and shipped to The University of Manchester, United Kingdom on cold blocks [see Figure S2 (Supporting Information) for starting soil moisture content]. Once in Manchester, the soils were sieved through 2 mm mesh and the permanent wilt point (PWP) was measured for each sample (based on Saxton and Rawls 2006). We calculated gravimetric soil moisture content for each soil sample by drying soils at 80°C for 48 hours and determining the proportional water loss. Preliminary soil moisture tests on the soils showed that in our experimental soils, only Latnjajaure dry heath soils were significantly drier in warmed than ambient soils, all others were relatively unchanged (Figure S2, Supporting Information). Each of the 48 samples were divided into four equal parts and placed in 50 ml Falcon tubes. This meant we had a matched pair of samples (one drought, one control) that would be destructively harvested at two time points (Figure S1, Supporting Information; days 0 and 56). The reason for having a separate Falcon tube to be harvested at each time point was to prevent disturbance to the microbial communities due for later harvest. The soils were kept in a growth chamber in the dark at 9°C, the average July temperature for both sites. Soils in the drought treatment were monitored as they dried down to the PWP, with the control maintained at 60% water holding capacity (WHC). When soil had reached PWP (9.17% for Latnjajaure, 6.93% for Val Bercla), the drought treatment commenced. All soils were maintained at either WHC (control) or PWP (drought) for 5 weeks, at which half of the microcosms were destructively harvested (day 0) to measure resistance of functional genes and GHG fluxes to drought. The Day 0 measurements were used to calculate resistance metrics (see the section ‘Statistical analysis’). The remaining droughted microcosms were rewetted to WHC and then WHC was maintained in the remaining microcosms, with weekly gas flux measures and harvests before the final tubes were harvested on day 56. These weekly harvest measures were used to calculate resilience (see the section ‘Statistical analysis’) on a week-by-week basis.

### GHG measurements

GHGs were sampled on each harvest day, immediately prior to harvest. The Falcon tubes were sealed using a suba-seal and Parafilm. Using a syringe, 5 ml of gas was taken from each tube at 0, 10, 20, and 30 minutes after sealing and deposited into individual vacuum-sealed exetainers. These were then analyzed on a gas chromatograph (GC; Agilent 7890B, Cheadle, UK). Fluxes of N_2_O, CH_4_, and CO_2_ were calculated over the 30 minute timeframe corrected for air temperature and headspace (Levy et al. 2011).

### DNA extraction and real-time PCR

DNA was extracted from each soil harvest using the DNeasy PowerSoil Kit (Qiagen, Manchester, UK), following the manufacturer’s instructions. As the soils ranged in organic matter content, 0.25 g of soil was extracted, as recommended for highly organic soils. DNA concentration and quality were assessed using a NanoDrop 2000 (Thermo Fisher Scientific), and was PCR amplified with 16S rRNA gene primers. Amplified DNA was shipped on ice blocks to the Hawkesbury Institute for the Environment, Western Sydney, Australia, where they were kept at −80°C until further analysis. Each sample was diluted to 2 ng µl^−1^, and quantitative PCR (qPCR) reactions were carried out on five genes: 16S rRNA for general bacteria, ITS for fungi, *acdS* for ACC deaminase (as a proxy for stress), *nosZII* for N_2_O reductase, and *pmoA* for CH_4_ monooxygenase. The primers were diluted to 1:30. Each 10 µl qPCR reaction consisted of 1 µl of template (2 ng), 2.5 µl water, 0.75 µl of each primer, and 5 µl of mastermix (Light Cycler® 480 Probes Master, Roche Diagnostics Ltd.). The *pmoA* qPCR required 2 µl of template (4 ng). A calibration curve was included for each gene from 10^9^ to 10^4^ gene copies. The samples were analyzed in duplicate on a 384-well plate on a Light Cycler [see Table S1 (Supporting Information) for details of primers and optimum annealing temperatures]. The amplification of the samples was analyzed using Light Cycler software.

### Statistical analysis

To ask if long-term warming increases functional gene abundances in response to drought, and if vegetation type is associated with a significant difference in gene abundance, we first standardized the functional genes *pmoA, nosZII*, and *acdS* by calculating the ratio with 16S rRNA genes. We built linear mixed effects models with experimental replicate (i.e. matched drought-control pairs) nested within origin field treatment (i.e. experimental warming or ambient temperature) as random factors, and three-way interactions between the fixed factors of timepoint, vegetation type, warming, and drought (Bates et al. 2013). Including a three-way interaction between our fixed effects allowed us to test if long-term warming increases gene abundance in response to drought, and if vegetation type modifies this response. We ran these models with functional gene abundances, and GHG fluxes as the response variables in turn. We used likelihood ratio deletion tests to determine whether the random effects improved model fit using the *MuMIn* package in R to find both marginal and conditional *R*^2^ values (Barton 2023, Nakagawa and Schielzeth 2013). Marginal *R*^2^ values calculate fixed effects only, while conditional effects consider both fixed and random effects. Where models were simplified these values would be identical. We used Tukey’s Honest Significant Difference to identify specific treatment effects (vegetation, drought, and warming). We also built these models for GHG emissions.

To ask if warming affects the resistance or resilience of functional gene abundances to drought, we calculated resistance and resilience of gene abundance after drought using the metrics of Orwin and Wardle (2004). Gene abundances and ratios were first standardized to the mean, then resistance was defined as the magnitude of the change in the variable caused by the treatment, and was calculated as RS= 1–2*|D_0_|*(C_0_+|D_0_|), where D_0_ is the difference between the well-watered control (C_0_) and the drought (P_0_) on Day 0, the last day of the drought treatment. Resilience by day 56 was calculated as RL= 2*|D_0_|*(|D_0_|+|D_x_|) – 1, where D_x_ is the difference between the well-watered control and the drought treatment on day 56. Drought against control pairs were compared for ambient soils, then warmed soils, so it was possible to detect whether resistance and resilience to drought were changed if there was a legacy of warming. The values are bounded between 0 and 1, where 0 is a 100% change from the control, 1 is full resistance or resilience (i.e. no change from the control), and negative numbers denote an increase of the values relative to the control, indicating an overshoot of the response variable. 95 % confidence intervals were calculated to explain whether there was a difference between the resistance and resilience of the gene abundances under drought and warming vs the day 0 control. Where the error bars overlapped with 1, there was no difference between the treatment in question and the day 0 control.

To test the second hypothesis (and partially third), that GHG emissions will be associated with functional microbial genes, and that this link will be modified by prior warming and a subsequent drought, we fit linear mixed effects models testing the response of each GHG to each experimental treatment. We used day 56 only (8 weeks of being maintained at field capacity) and included warming, drought, vegetation type and the genes we expected to play roles in the flux. For each GHG, we repeated the model described above including each single gene in turn. We included all possible three-way interaction terms between vegetation type, drought, warming, and the single gene (for 16S rRNA gene and ITS) or gene ratio (for *pmoA, nosZII*, and *acdS*), to reduce the potential for Type I error caused by multiple testing. Four-way interactions are shown to be difficult to interpret and have low explanatory power (Leuzinger et al. 2011). For each model, we included experimental replicate (i.e. matched drought-control pairs) nested within origin field treatment (i.e. experimental warming or ambient temperature) as nested random factors. These were simplified for random effects as before.

For CO_2_ flux (respiration) and CH_4_, we fit linear mixed effects models with vegetation type, warming and drought as factor level explanatory variables, and one gene for each model: 16S rRNA genes, ITS, *pmoA*:16S rRNA, and *acdS*:16S rRNA genes. For N_2_O *pmoA*:16S rRNA genes were substituted for *nosZII*:16S rRNA genes. The model was initially simplified by testing the importance of the nested random effects to conserve degrees of freedom using likelihood ratio deletion tests, but was not simplified further. We fit all models using *lmer* in R version 4.0.3 (Bates et al. 2013, R Development Team 2020). All code can be found in the supplementary material.

## Results

### Treatment effects on functional genes

**Figure 2.**
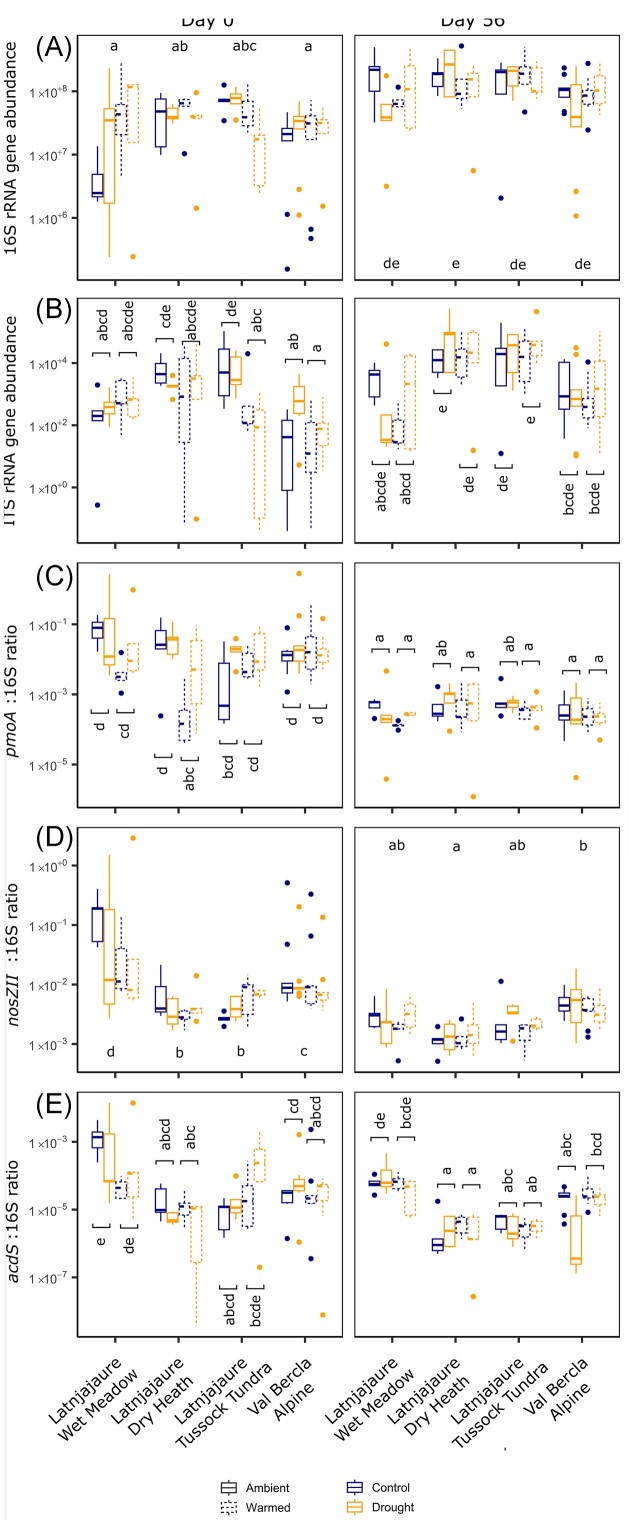
Gene abundances by treatment on day 0, immediately after the drought, and day 56, after 7 weeks of rewetting. Groups that varied significantly are displayed using horizontal lines and significance stars. Significance as follows: *P < .05, **P < .01, and ***P < .001. (A) Bacteria (16S): Timepoint F_1,123_ = 67.52, *P* < .001, Vegetation type F_3,123_ = 3.55, *P* = .023, (B) fungus (ITS): Timepoint × Vegetation type × Warming: F_3,123_ = 3.34, *P* = .021, (C) *pmoA*: Timepoint × Vegetation type × Warming: F_3,163_ = 3.23, *P* = .024 D) *nosZ:* Timepoint × Vegetation type: F_3,122_ = 10.28, *P* < .001 E) *acdS*: Timepoint × Warming: F_1,163_ = 3.99, *P* = .047, Vegetation type × Warming: F_3,163_ = 3.03, *P* = .031. There were no significant effects of drought and so these have not been included here for clarity.

When we analyzed the response of the functional genes between days 0 and 56, we found no significant effect of drought at all, although there were significant effects of warming and varying responses under different vegetation types (Fig. 2). For the raw gene abundances, we only observed a timepoint and vegetation effect (Fig. 2A; Timepoint F_1,123_ = 67.52, *P* < .001, Vegetation type F_3,123_ = 3.55, *P* = .023), where on day 0 there was a decrease in 16S rRNA gene from wetter, more C-rich soils (Latnjajaure wet meadow) to drier, less C-rich soils (Val Bercla alpine). The raw values for the genes in day 56 showed an increase in 16S rRNA gene compared with day 0 for all soils, and a decrease in all gene ratios, but no strong or consistent treatment effects (Fig. 2).

**Figure 3.**
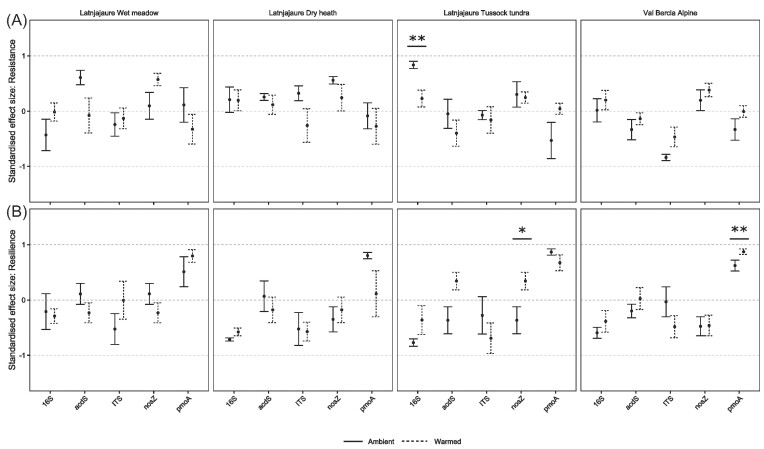
Resistance and resilience metrics of functional gene abundances. The warmed and ambient treatments are calculated as the droughted abundance against the control abundance for their respective warming treatment. It is then apparent if warming alters the level of gene abundance change in response to drought. Here, there is little evidence of resistance or resilience in any gene. (A) Resistance of microbial functional genes at the end of the drought, comparing the effect sizes of drought on ambient (solid) and warmed (dashed) soils. (B) Resilience of microbial functional genes 56 days after the end of the drought. Significance stars represent a significant difference between warmed and unwarmed treatments. Error bars depict confidence intervals at 95%. If the error bar crosses 0, this indicates a 100% change in value from the control, meaning no resistance. If the values are negative, the drought treatment yielded an increase in the functional gene compared with the control. Significance stars denote a significant difference between warmed and unwarmed soil (* *P* < .05, ** *P* < .01, and *** *P* < .001).

When we calculated resistance and resilience, we found more nuanced responses to the drought. The legacy of soil warming did not affect the resistance of functional gene abundance to drought on day 0 (Fig. 3A). None of the genes we examined were resistant to drought, evidenced by a lack of overlap of confidence intervals with 1. Only the 16S rRNA gene showed a significant response to warming, which was more resistant to drought in warmed than ambient tussock tundra treatment plots (F_1,8_ = 12.18, *P* = .008). No genes showed resilience to drought, as gene abundances did not recover to the level of the control after 56 days recovery time (as there was no overlap of the bars with 1; Fig. 3B). In the Latnjajaure wet meadow, the resilience values of all five genes were near zero, indicating no improvement over the recovery period. For the other vegetation types, resilience of gene abundances were mostly negative, indicating that the abundances were higher on day 56 relative to the control, and thus signifying a loss of resilience. There was a significant effect of warming on *nosZII* gene resilience in tussock tundra, where warmed soils showed evidence of gene abundances returning to the control values. There was no recovery in ambient temperatures, as resilience still showed increased abundance relative to the control on day 56, so there was no true recovery. In the alpine vegetation type, warmed soils showed higher resilience postdrought than soils of the ambient plots, as shown by a significant effect of warming on resilience of *pmoA* genes.

### Treatment effects on GHG fluxes

**Figure 4.**
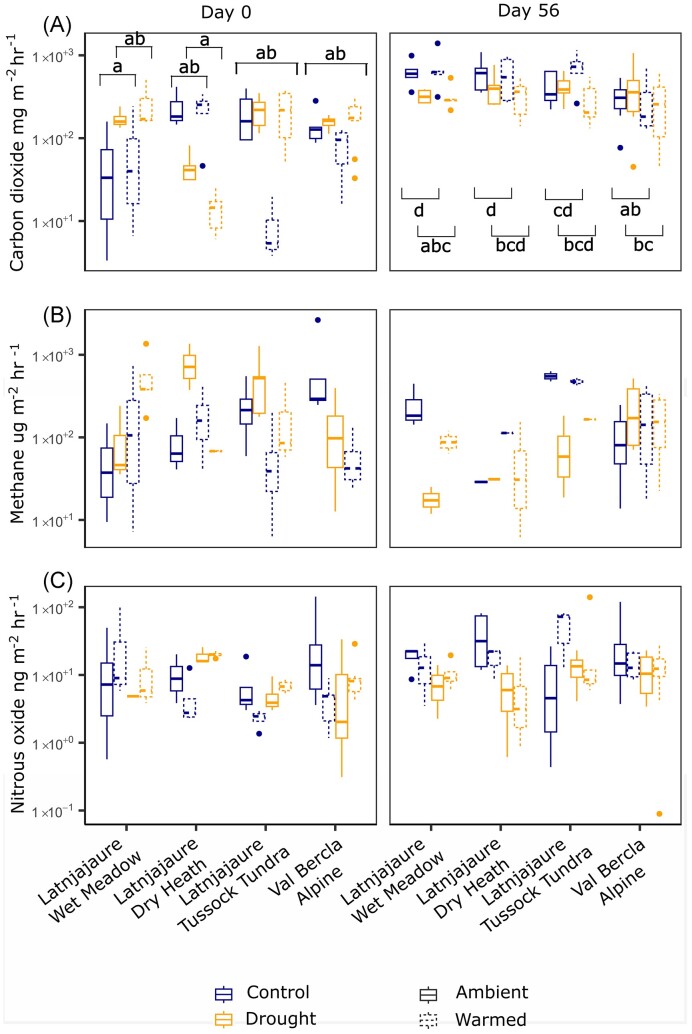
Treatment effects on GHG emissions on day 0 immediately after the drought, and day 56, which is 56 days after rewetting commenced. Treatment effects are displayed on the graph, with brackets denoting the groups that are significantly different from one another. Significance as follows: *P < .05, **P < .01, and ***P < .001. (A) CO_2_ flux Time × Vegetation Type × Drought: F_3,163_ = 4.51, *P* = .005, (B) CH_4_ flux Time × Vegetation Type × Warming: F_3,163_ = 3.13, *P* = .028, (C) N_2_O flux Time (F_1,63_ = 4.48, *P* = .036), Drought × Warming (F_1,163_ = 3.93, *P* = .049).

CO_2_ gas fluxes showed a significant interaction between timepoint, drought and vegetation type, but no effect of warming (Fig. 4A; F_3,163_ = 4.51, *P* = .005). This meant all fluxes were higher on day 56 than day 0, and while there was a weak drought effect in most vegetation types, there was a significant decrease in droughted soils from the wet meadow on day 56, as shown by *post hoc* tests. For CH_4_ fluxes, there was no significant effect of drought, but we observed a three-way interaction between timepoint, warming and vegetation type (Fig. 4B; F_3,163_ = 3.13, *P* = .028). On day 0, all vegetation types showed lower CH_4_ in warmed soils except the wet meadow. On day 56, warmed soils led to higher CH_4_ fluxes than ambient, again apart from the wet meadow. For N_2_O, we observed a time effect, where fluxes were slightly higher on day 56 than day 0, and an interaction between drought and warming, but no vegetation effect (Fig. 4C; Time F_1,63_ = 4.48, *P* = .036, Drought × Warming F_1,163_ = 3.93, *P* = .049).

### GHGs are linked to functional gene abundances

**Figure 5.**
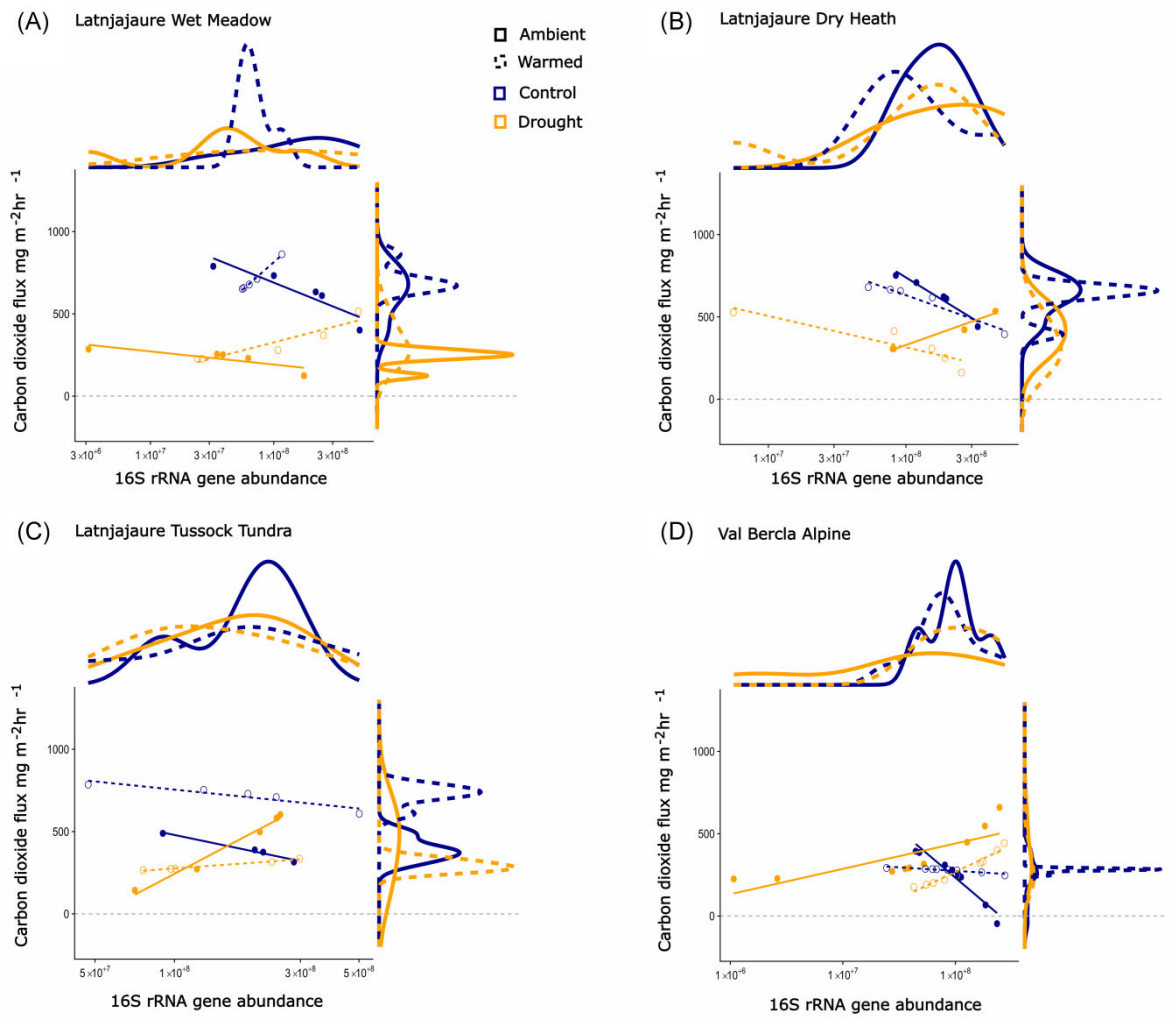
Flux of CO_2_ influenced by vegetation type (F_3,67_ = 4.35, *P* = .007), and drought × warming × bacteria (16S) gene abundance: F_1,67_ = 4.69, *P* = .034, *R*^2^ = 0.38. Data presented is measured on day 56 of recovery from drought. (A) Latnjajaure wet meadow, (B) Latnjajaure dry heath, (C) Latnjajaure tussock tundra, and (D) Val Bercla alpine. Values shown here are predicted values from the mixed effects models, and density graphs are presented for each axis for clarity.

Following the 56-day recovery period, a set of complex interactions between functional genes and the experimental treatments influenced CO_2_ emissions. 16S rRNA bacterial gene abundance impacted CO_2_ flux via a three-way interaction with warming and drought, and this varied across vegetation types (Fig. 5). Warming alone resulted in both positive and negative effects on bacterial associations with CO_2_ flux, while drought alone increased CO_2_ with increased 16S rRNA gene in all vegetation types. The mixed results here meant that soils that were both warmed and subjected to drought generally had a lower CO_2_ flux, which was positively associated with bacterial abundance in all vegetation types except dry heath.

**Figure 6.**
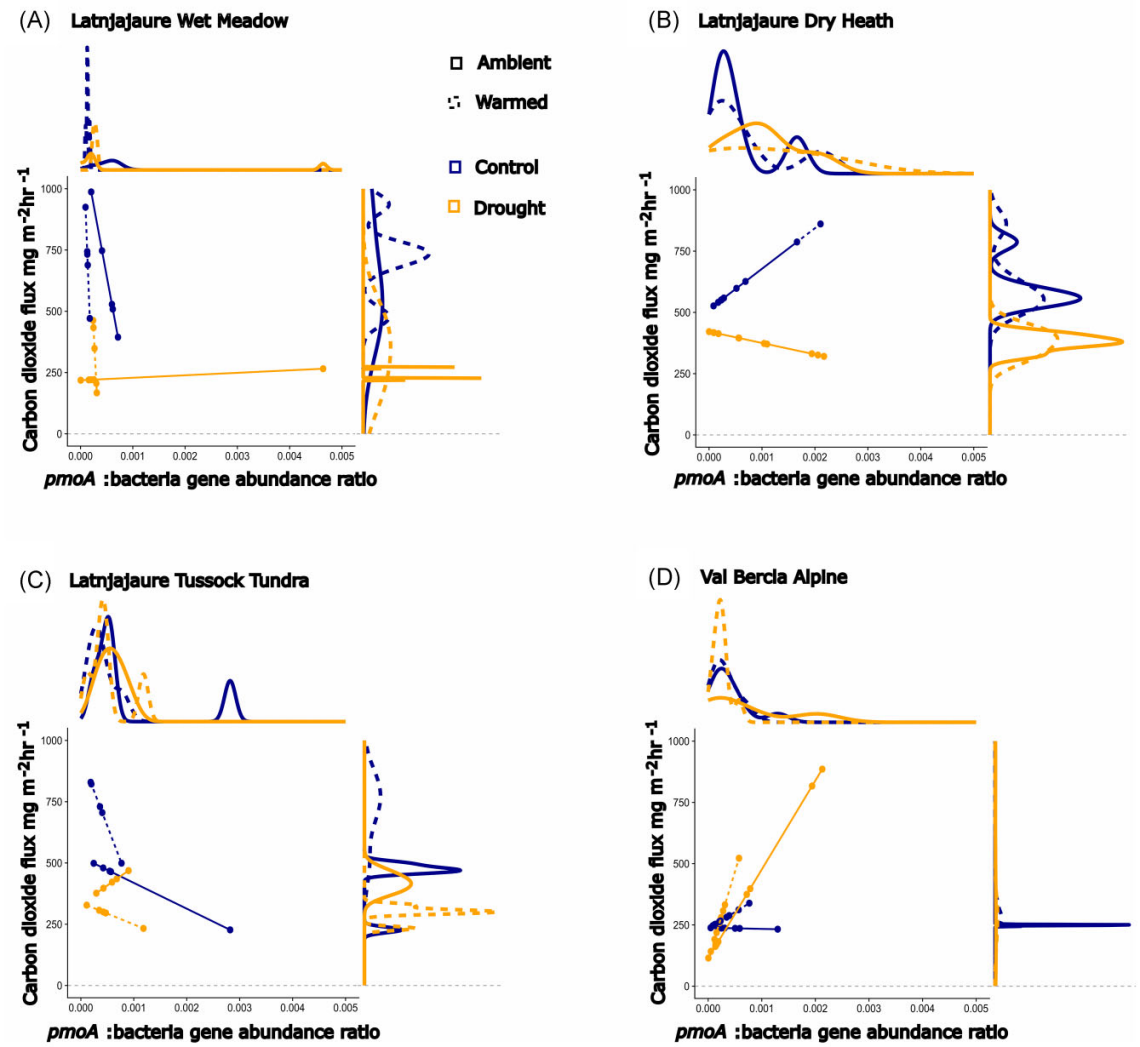
Flux of CO_2_ influenced by an interaction between vegetation type, *pmoA*: 16S bacterial ratio and drought (F_3,67_ = 3.13, *P* = .031, *R*^2^ = 0.44). Data presented is measured on day 56 of recovery from drought. (A) Latnjajaure wet meadow, (B) Latnjajaure dry heath, (C) Latnjajaure tussock tundra, and (D) Val Bercla alpine. Values shown here are predicted values from the mixed effects models, and density graphs are presented for each axis for clarity.

*pmoA*:16S rRNA gene abundance ratios was positively associated with CO_2_ fluxes, with a significant effect of drought in vegetation types from wetter soils (Fig. 6A and B). We observed no effect of prior warming. In wet meadow and tussock tundra, increased *pmoA*:16S rRNA gene ratio decreased CO_2_ in control soils while under drought, *pmoA* did not influence CO_2_ flux (Fig. 6A and C). In dry heath soils, *pmoA*:16S rRNA gene positively correlated with CO_2_ in control soils, and negatively correlated with CO_2_ in droughted soils (Fig. 6B). In alpine soils, *pmoA*:16S rRNA gene ratio was associated with slightly increased CO_2_ flux in control soils, and strongly increased CO_2_ in droughted soils.

We did not detect significant effects of prior warming or drought on the relationship between *pmoA:*16S rRNA gene and CH_4_ flux, although there was a significant interaction between vegetation type and *pmoA:*16S rRNA gene (F_3,67_ = 3.11, *P* = .032). Here, we found that for all vegetation types except wet meadow, CH_4_ emissions, and ultimately CH_4_ uptake, were reduced with an increase in the *pmoA:*16S rRNA gene ratio. In the wet meadow, warming, drought, and vegetation type did not influence CH_4_ flux.

**Figure 7.**
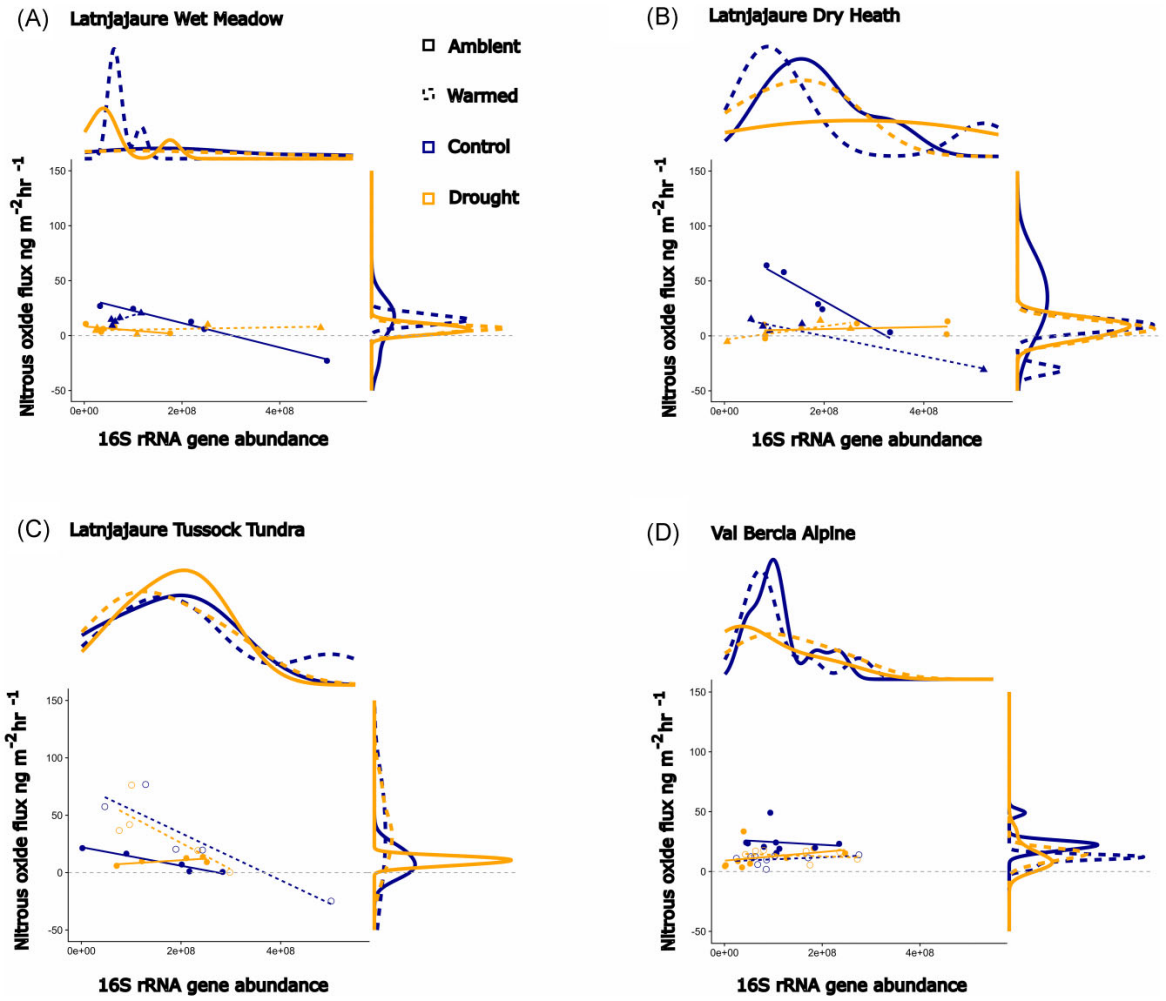
N_2_O gas flux affected by a main effect of 16S gene abundance (F_1,67_ = 7.13, *P* = .010), and an interaction between vegetation type and warming (F_3,67_ = 3.48, *P* = .021, conditional *R*^2^ = 0.51, marginal *R*^2^ = 0.30). Data presented is measured on day 56 of recovery from drought. (A) Latnjajaure wet meadow, (B) Latnjajaure dry heath, (C) Latnjajaure tussock tundra, and (D) Val Bercla alpine. Values shown here are predicted values from the mixed effects models, and density graphs are presented for each axis for clarity.

For N_2_O fluxes, there was a consistent relationship between the 16S rRNA gene, warming and vegetation type, although we observed no effect of drought (Fig. 7). Bacterial gene abundance was negatively associated with N_2_O flux, although the interaction between vegetation type and warming meant that for wet meadow and tussock tundra, warming increased N_2_O emissions, while for dry heath and alpine, warming decreased N_2_O (Fig. 7B and D). There was no association between N_2_O fluxes and the *nosZII* gene, the *acdS* gene, or the ITS fungal rRNA gene abundance (Table S2, Supporting Information).

## Discussion

In this study, we used soil from a series of long-term open-top chamber experiments to test whether long-term warming mediated the resistance and resilience of microbial functional gene abundances involved in mitigating GHG emissions to extreme drought. We further asked whether this has net consequences for GHG fluxes. Our first hypothesis was that long term warming will increase functional gene abundances involved in GHG emissions, and that these would have lower resistance, but greater resilience to drought than genes from unwarmed soils. While our findings did not support this hypothesis, they provide some support for our second hypothesis that GHG emissions are higher under warming and drought, although responses varied with the vegetation type the soils were derived from. We also found complex interactions between warming and drought affecting GHG emissions, showing that the introduction of strong climate perturbations may disrupt the link between functional genes and GHG emissions. In particular, we found strong support for the third hypothesis, and here showed that vegetation type has a large modulating role in warming and drought effects on both functional genes and GHGs, with forb-rich, wetter soils being more affected by long-term warming and subsequent drought.

We hypothesized that because of the cold, wet conditions in our study sites, leading to metabolic constraints on microbes, long-term warming would increase microbial functional gene abundances, and that this would be further increased by drought. This would lead to a loss of resistance to drought. We further expected a rapid recovery of gene abundances from drought in soils with a legacy of warming. In our study sites, temperatures are low, and in tussock tundra and wet meadow, waterlogging is common (Molau 2010, Scharn et al., 2021). These two factors tend to slow microbial growth and activity (Freeman et al. 2001). However, in our study, similarly to that of Jeanbille et al. (2021), we did not observe any change in gene abundance in response to drought, while warming effects occurred in ITS, *pmoA* and *acdS*. ITS gene abundance was increased under warming in the wet meadow, but decreased by warming in tussock tundra. The growth and extracellular signalling required for metabolic activity are energetically costly and occur more slowly in cold soils (Solly et al. 2017). However, there has been some question over whether fungal growth in response to warming occurs due to the decline in soil moisture that often occurs in tandem with warming (Rudgers et al. 2014). Here, we show that this is not the case: responses of fungi to warming and drought were independent, but there is a legacy effect from the dominant plant species and/or soil type. This is consistent with findings from Rudgers et al. (2014), who showed that in lower moisture soil conditions, arbuscular mycorrhizal fungi colonized plants more readily, but not in soils that are solely warmed. In our study, drought was imposed on bare soil under laboratory conditions in the absence of plants, so any shift derived from the vegetation type would have occurred through the legacy impact of the different plant communities on the soil properties through factors such as residues.

The gene that encodes the CH_4_-oxidizing enzyme CH_4_ monooxygenase, *pmoA*, increased immediately after drought across all vegetation types except for the alpine. While some previous studies have recorded an increase in *pmoA* abundance after experimental warming (Yue et al. 2015), a recent meta-analysis found little evidence of this gene responding to warming across a range of ecosystem types (Liu et al. 2019b). *pmoA* is one of a number of genes that encode enzymes that consume CH_4_, including *mmoX*. However, *mmoX* is found in a low number of bacterial strains and is often not detected in sufficient numbers to be able to infer any effect of climate change drivers (Gupta et al. 2012, Unger et al. 2021). Others have observed increased activity of methanotrophic bacteria as soils dry because drought enables CH_4_ and oxygen to be diffused into the soil (Ran et al. 2017). Under drought, microbes often increase in activity, particularly when soils were previously waterlogged, and so an increase in abundance of *pmoA* gene could be expected. In a similar system (tussock tundra in Latnjajaure), Keuschnig et al. (2022) showed that methanogens were negatively impacted by drought, leading to a net decrease in CH_4_ production. The organic C content of the soil has been suggested to only weakly effect CH_4_ emissions (Xu et al. 2020b). Thus, as we did see a change in *pmoA* abundance across vegetation type, it is likely to be linked with water availability and soil drainage properties, influenced by vegetation type.

We further expected that a legacy of long-term warming would reduce the resistance but increase the resilience of the microbial community to a severe drought, and that we could use abundance of functional genes in the soil as a proxy for this, as gene expression may be up- or down-regulated in response to abiotic conditions. Our results indicate that a legacy of warming was acting on microbial gene abundance, but it did not clearly influence the response of gene expression to drought. In general, we observed an increase in *pmoA* and *acdS* genes after drought, as expected, as *pmoA* is associated with aerobic conditions (Knief 2015), while *acdS* is presumed to be expressed by microbes in response to stress (Singh et al. 2015). Further, we observed stronger effects of warming on microbial drought resistance in the Oro-Arctic soils compared with the alpine soils. It is possible that this is due to the larger warming effect of the OTCs, but even then, the effect of warming on *pmoA* and *acdS* was inconsistent. Many studies have observed relatively weak effects of warming on the soil microbial community and associated functions (Li et al. 2020, Sarneel et al. 2020). A common finding is that simulated drought and warming only affected the microbial community structure when both treatments were in combination (Zhang et al. 2015). By day 56, we expected that they would have reduced back to control levels, but it appeared that after 7 weeks of rewetting the functional genes had increased in abundance relative to the control. This was irrespective of the number or type of global change factors applied, suggesting that both warming and drought leads to a release of abiotic constraints on microbial gene expression. This effect is likely to be linked with pulses of microbial activity and subsequent mineralization of N and C, known as the ‘Birch effect’ (Birch 1958).

Our second hypothesis was that GHG emissions would be linked with functional gene abundance, and that long-term warming would increase net GHG flux through increased microbial activity. We further anticipated that drought would increase GHG flux further through exposing decomposable substrates and increasing oxygen availability. In Oro-Arctic tundra and alpine regions, there is potential for changes in abiotic conditions to result in rapid increases in GHGs through the decomposition of previously inaccessible organic matter (Voigt et al. 2017). Drying and warming of soils that are often waterlogged or frozen can result in strong and long-lasting changes in net GHG emissions (Günther et al. 2020). In our study, we saw that vegetation type, genes, warming and drought were all important factors contributing to changes in GHG emissions, with the most important factor being vegetation type. Interestingly, one suggestion is that warming increases the competition between plants and fungi for N in these alpine or Oro-Arctic soils, which may mask the response of fungi (Xiong et al. 2014). Given that for most genes there was little effect of the warming and drought treatments, we can infer that these treatments impact emissions through a more complex set of mechanisms than release of constraints caused by low temperatures. It is likely that warming and drought has increased accessibility of formerly unavailable substrates, leading to increased activity and metabolism through release of the enzymatic latch (Freeman et al. 2001). This is particularly true for CO_2_, which in our study had complex interactions with the treatments and bacteria. However, the strong positive effect of bacterial 16S rRNA gene abundance on GHG fluxes suggests that while bacterial abundances were not altered by the treatments, bacterial activity was. In particular, the warming effect compounded the drought effect, leading to a reduction in CO_2_ and loss of the bacterial signal in the data. By removing the plants in our study, we have provided strong evidence of a significant relationship between bacterial abundance and CO_2_ emissions under abiotic stress. N_2_O emissions occur through a primarily anaerobic pathway (Prosser et al. 2020), and so by droughting and warming the soil, leading to aeration, it is possible that the pathway was interrupted in our study.

Our third hypothesis was that vegetation type, and the associated edaphic characteristics, are likely to result in different gene and GHG responses to drought and warming. Specifically, we expected soils from wetter more forb-rich areas to be more responsive to the climate change treatments. Wetter soils were more C rich, and we demonstrated a simple gradient of soil moisture, C and N from Latnjajaure wet meadow, at the wet C rich end, to Val Bercla alpine, at the dry, C poor end. We expected to observe decreasing effect sizes as soils became drier and C poor. Contrary to this hypothesis, we found that the gene that reduced N_2_O, *nosZII*, was only affected by vegetation type, with a clear increase in the gene in drier alpine areas compared to the wetter forb-rich areas and not by warming and drought. We also found that CO_2_ fluxes were strongly influenced by vegetation type, being higher in the dry heath than the alpine, which could be due to the differing inputs by plant species (Kuzyakov and Gavrichkova 2010). Dwarf shrubs such as those dominant in the dry heath (e.g. *C. tetragona*) are known to add more polyphenolic compounds to the soil than other plant types, which can inhibit microbial activity (Ward et al. 2013). While the drought was ongoing (day 0), droughted soils from dry heath communities had lower CO_2_ fluxes than soils from grasses or forb-based communities. After rewetting, the dry heath soils had higher fluxes than the other vegetation types, which could indicate that these are less stable and more susceptible to C losses during climate perturbations. However, the other GHGs (CH_4_ and N_2_O) were highly inconsistent across vegetation type. This may be because there is no clear direct link between plants and CH_4_ or N_2_O emissions, which are mainly driven by specialized microbial activities. Our study is an *in vitro* look at the response of gene abundances to abiotic stress, and as such the common explanations for observed trends, e.g. changes in plant metabolism, root exudation and litter decomposition, do not apply here (Hammerl et al. 2019). Because our experiment took place in microcosms, the effects seen would be due to a legacy of the plant species, and the soil type, which could take the form of partially decomposed plant tissues, root exudates, or potentially changes in soil structure.

## Conclusions

Our findings suggest that across Oro-Arctic and alpine sites, long-term increases in temperature, combined with extreme drought events, could lead to changes in functional gene abundances, and that these responses are closely linked with vegetation type. Specifically, our findings suggest that wetter ecosystems, with forb-dominated vegetation and C-rich soil, are most vulnerable to individual and combined effects of long-term warming and drought. Previous research on highly organic soils in Oro-Arctic and alpine soils points to strong and long-lasting responses of microbial communities, characterized by functional gene abundances, to climate change. Our study, which was carried out on more mineral soils shows a similar result. Soil microbial communities in very cold or waterlogged soils are not resilient to changes in soil temperature or water availability. However, in most cases, the effect on GHG fluxes may be less than expected. Taken together, our results indicate that while individual microbial functional genes may be resistant to warming and drying, there could be shifts in GHG emissions through altered microbial gene expression.

## Supplementary Material

fiad145_Supplemental_Files

## References

[bib1] Bartón K . Mumin: Multi-model Inference. R Package Version 1.47.5. CRAN, 2023.

[bib2] Bates D , Maechler M, Bolker B et al. lme4: Mixed-effects model using Eigen and S4. R Package Version 1.0-4. CRAN, 2013.

[bib3] Birch HF . The effect of soil drying on humus decomposition and nitrogen availability. Plant Soil. 1958;10:9–31.

[bib7] Commane R , Lindaas J, Benmergui J et al. Carbon dioxide sources from Alaska driven by increasing early winter respiration from Arctic tundra. Proc Nat Acad Sci USA. 2017:114:5361–6. 28484001 10.1073/pnas.1618567114 PMC5448179

[bib8] Freeman C , Ostle N, Kang H. An Enzymic “Latch” on a global carbon store: a shortage of oxygen locks up carbon in peatlands by restraining a single enzymes. Nature. 2001;409:149. 10.1038/35051650 11196627

[bib9] García Criado M , Myers-Smith IH, Bjorkman AD et al. Woody plant encroachment intensifies under climate change across Tundra and Savanna biomes. Global Ecol Biogeogr. 2020;29:925–43.

[bib10] Grabherr G , Gottfried M, Pauli H. Climate change impacts in alpine environments. Geogr Compass. 2010;4:1133–53.

[bib11] Günther A , Barthelmes A, Huth V et al. Prompt rewetting of drained peatlands reduces climate warming despite methane emissions. Nat Commun. 2020;11:1644. 32242055 10.1038/s41467-020-15499-z PMC7118086

[bib12] Gupta S , Pandey S. Unravelling the biochemistry and genetics of ACC deaminase – an enzyme alleviating the biotic and abiotic stress in plants. Plant Gene. 2019;18:100175.

[bib13] Gupta V , Smemo KA, Yavitt JB et al. Active methanotrophs in two contrasting North American peatland ecosystems revealed using DNA-SIP. Soil Microbiol. 2012;63:438–45. 10.1007/s00248-011-9902-z 21728037

[bib14] Hagedorn F , Gavazov K, Alexander JM. Above- and belowground linkages shape responses of mountain vegetation to climate change. Science. 2019;365:1119–23. 31515385 10.1126/science.aax4737

[bib15] Hammerl V , Kastl EM, Schloter M et al. Influence of rewetting on microbial communities involved in nitrification and denitrification in a grassland soil after a prolonged drought period. Sci Rep. 2019;9:2280. 30783152 10.1038/s41598-018-38147-5 PMC6381133

[bib16] Hartley IP , Garnett MH, Sommerkorn M et al., A potential loss of carbon associated with greater plant growth in the European Arctic. Nat Clim Change. 2012;2:875–9.

[bib72_1700203413707] Henry GHR , Hollister RD, Klanderud K et al. The International Tundra Experiment (ITEX): 30 years of research on tundra ecosystems. Arctic Science. 2022;8.

[bib17] Henry GHR , Molau U. Tundra plants and climate change: the International Tundra Experiment (ITEX). Global Change Biol. 1997;3:1–9.

[bib18] Hollister RD , Elphinstone C, Henry GHR et al. A review of Open Top Chamber (OTC) performance across the ITEX Network. Arctic Sci. 2022;9:345–55.

[bib58] IPCC , Climate change 2023: synthesis report. Contribution of Working Groups I, II and III to the Sixth Assessment Report of the Intergovernmental Panel on Climate Change. In: Core Writing Team, Lee H, Romero J (eds), Geneva: IPCC, 2023, 35–115. 10.59327/IPCC/AR6-9789291691647.

[bib19] Jenabille M , Clemmensen K, Juhanson J et al. Site-specific responses of fungal and bacterial abundances to experimental warming in litter and soil across Arctic and alpine tundra. Arctic Sci. 2021;8:992–1003.

[bib20] Keuschnig C , Larose C, Rudner M et al. Reduced methane emissions in former permafrost soils driven by vegetation and microbial changes following drainage. Global Change Biol. 2022;28:3411–25. 10.1111/gcb.16137 PMC9314937 35285570

[bib21] Knief C . Diversity and habitat preferences of cultivated and uncultivated aerobic methanotrophic bacteria evaluated based on pmoA as molecular marker. Front Microbiol. 2015;6:1346. 26696968 10.3389/fmicb.2015.01346 PMC4678205

[bib22] Kuzyakov Y , Gavrichkova O. REVIEW: time lag between photosynthesis and carbon dioxide efflux from soil: a review of mechanisms and controls. Global Change Biol. 2010;16:3386–406.

[bib23] Lafuente A , Durán J, Delgado-Baquerizo M et al. Biocrusts modulate responses of nitrous oxide and methane soil fluxes to simulated climate change in a Mediterranean dryland. Ecosystems. 2020;23:1690–701. https://doi.or/10.1007/s10021-020-00497-5.

[bib24] Leuzinger S , Luo Y, Beier C et al. Do global change experiments overestimate impacts on terrestrial ecosystems?. Trends Ecol Evol. 2011;26:236–41. 21444122 10.1016/j.tree.2011.02.011

[bib25] Levy PE , Gray A, Leeson SR et al. Quantification of uncertainty in trace gas fluxes measured by the static chamber method. Eur J Soil Sci. 2011;62:811–21.

[bib26] Li L , Zheng Z, Wang W et al. Terrestrial N_2_O emissions and related functional genes under climate change: a global meta-analysis. Global Change Biol. 2020;26:931–43. 10.1111/gcb.14847 31554024

[bib27] Liu H , Khan MY, Carvalhais LC et al. Soil amendments with ethylene precursor alleviate negative impacts of salinity on soil microbial properties and productivity. Sci Rep. 2019a;9:6892. 31053834 10.1038/s41598-019-43305-4 PMC6499801

[bib28] Liu J , Chen H, Zhu Q et al. A novel pathway of direct methane production and emission by eukaryotes including plants, animals and fungi: an overview. Atmos Environ. 2015;115:26–35.

[bib29] Liu L , Estiarte M, Peňuelas J. Soil moisture as the key factor of atmospheric CH_4_ uptake in forest soils under environmental change. Geoderma. 2019b;355:113920.

[bib30] Loganathan P , Myung H, Muthusamy G et al. Effect of heavy metals on acdS gene expression in *Herbaspirillium* sp. GW103 isolated from rhizosphere soil. J Basic Microbiol. 2015;55:1232–8. 25903936 10.1002/jobm.201500008

[bib31] Marion GM , Henry GHR, Freckman DW et al. Open-top designs for manipulating field temperature in high-latitude ecosystems. Global Change Biol. 1997;3:20–32.

[bib32] Martins CSC , Nazaries L, Delgado-Baquerizo M et al. Identifying environmental drivers of greenhouse gas emissions under warming and reduced rainfall in boreal-temperate forests. Funct Ecol. 2017;31:2356–68.

[bib34] Molau U . Long-term impacts of observed and induced climate change on Tussock Tundra near its Southern limit in Northern Sweden. Plant Ecolog Divers. 2010;3:29–34.

[bib33] Molau U . Responses to natural climatic variation and experimental warming in two Tundra plants with contrasting life forms: *Cassiope tetragona* and *Ranunculus nivalis*. Global Change Biol. 1997;3:97–107.

[bib35] Nakagawa S , Schielzeth H. A general and simple method for obtaining R2 from generalized linear mixed-effects models. Methods Ecol Evol. 2013;4:133–42.

[bib36] Nikolic B , Schwab H, Sessitsch A. Metagenomic analysis of the 1-Aminocyclopropane-1-Carboxylate Deaminase Gene (acdS) operon of an uncultured bacterial endophyte colonizing Solanum tuberosum L. Arch Microbiol. 2011;193:665–76. 21523387 10.1007/s00203-011-0703-z

[bib37] Olefeldt D , Turetsky MR, Crill PM et al. Environmental and physical controls on northern terrestrial methane emissions across permafrost zones. Global Change Biol. 2013;19:589–603. 10.1111/gcb.12071 23504795

[bib38] Orellana LHR , Rodriguez LM et al. Detecting nitrous oxide reductase (nosZ) genes in soil metagenomes: method development and implications for the nitrogen cycle. mBio. 2014;5:3. 10.1128/mBio.01193-14 PMC4049103 24895307

[bib39] Orhan F . Alleviation of salt stress by Halotolerant and Halophilic plant growth-promoting bacteria in wheat (*Triticum aestivum*). Braz J Microbiol. 2016;47:621–7. 27133557 10.1016/j.bjm.2016.04.001 PMC4927673

[bib40] Orwin KH , Wardle DA. New indices for quantifying the resistance and resilience of soil biota to exogenous disturbances. Soil Biol Biochem. 2004;36:1907–12.

[bib41] Palomo I . Climate change impacts on ecosystem services in high mountain areas: a literature review. Mount Res Dev. 2017;37:179–87.

[bib42] Prosser JI , Hink L, Gubry-Rangin C et al. Nitrous oxide production by ammonia oxidisers: physiological diversity, niche differentiation and potential mitigation strategies. Glob Change Biol. 2020;26:103–18. 10.1111/gcb.14877 31638306

[bib73_1700203490578] R Core Team . R: A language and environment for statistical computing. R Foundation for Statistical Computing. Vienna, Austria, 2020. http://www.R-project.org/.

[bib43] Ran Y , Xie J, Xu X. et al. Warmer and drier conditions and nitrogen fertilizer application altered methanotroph abundance and methane emissions in a vegetable soil. Environ Sci Pollut Res Int. 2017;24:2770–80. 27837471 10.1007/s11356-016-8027-9

[bib44] Rantanen M , Karpechko AY, Lipponen A et al. The Arctic has warmed nearly four times faster than the globe since 1979. Commun Earth Environ. 2022;3:168.

[bib45] Rudgers JA , Kivlin SN, Whitney KD et al. Responses of high-altitude graminoids and soil fungi to 20 years of experimental warming. Ecology. 2014;95:1918–28. 25163124 10.1890/13-1454.1

[bib46] Sarneel JM , Sundqvist MK, Molau U et al. Decomposition rate and stabilization across six Tundra vegetation types exposed to >20 years of warming. Sci Total Environ. 2020;724:138304. 32408462 10.1016/j.scitotenv.2020.138304

[bib47] Saxton KE , Rawls WJ. Soil water characteristic estimates by texture and organic matter for hydrologic solutions. Soil Sci Soc Am J. 2006;70:1569–78.

[bib48] Scharn R , Brachmann CG, Patchett A et al. Vegetation responses to 26 years of warming at Latnjajaure field station, Northern Sweden. Arctic Sci. 2022;8:1026–39.

[bib49] Scharn R , Little CJ, Bacon CD et al. Decreased soil moisture due to warming drives phylogenetic diversity and community transitions in the tundra. Environ Res Lett. 2021;16:064031.

[bib50] Seo J , Jang I, Jung JY et al. Warming and increased precipitation enhance phenol oxidase activity in soil while warming induces drought stress in vegetation of an Arctic ecosystem. Geoderma. 2015;259-260:347–53.

[bib51] Sheik CS , Beasley WH, Elshahed MS et al. Effect of warming and drought on grassland microbial communities. ISME J. 2011;5:1692–700. 21451582 10.1038/ismej.2011.32 PMC3176507

[bib52] Shine KP , Fuglestvedt JS, Hailemariam K et al. Alternatives to the global warming potential for comparing climate impacts of emissions of greenhouse gases. Clim Change. 2005;68:281–302.

[bib53] Singh BK , Bardgett RD, Smith P et al. Microorganisms and climate change: terrestrial feedbacks and mitigation options. Nat Rev Microbiol. 2010;8:779–90. 20948551 10.1038/nrmicro2439

[bib54] Singh RP , Shelke GM, Kumar A et al. Biochemistry and genetics of ACC deaminase: a weapon to “stress ethylene” produced in plants. Front Microbiol. 2015;6:937. 26441873 10.3389/fmicb.2015.00937 PMC4563596

[bib55] Solly EF , Lindahl BD, Dawes MA et al. Experimental soil warming shifts the fungal community composition at the alpine treeline. New Phytol. 2017;215:766–78. 28543616 10.1111/nph.14603

[bib56] Stenström M , Gugerli F, Henry GHR. Response of *Saxifraga oppositifolia* L., to simulated climate change at three contrasting latitudes. Global Change Biol. 1997;3:44–54.

[bib57] Szymański W . Quantity and chemistry of water-extractable organic matter in surface horizons of Arctic soils under different types of Tundra vegetation – a case study from the Fuglebergsletta coastal plain (SW Spitsbergn). Geoderma. 2017;305:30–9.

[bib59] Tveit AT , Urich T, Frenzel P et al. Metabolic and trophic interactions modulate methane production by Arctic peat microbiota in response to warming. Proc Nat Acad Sci USA. 2015;112:E2507–16. 25918393 10.1073/pnas.1420797112 PMC4434766

[bib60] Unger V , Liebner S, Koebsch F et al. Congruent changes in microbial community dynamics and ecosystem methane fluxes following natural drought in two restored fens. Soil Biol Biochem. 2021;160:108348.

[bib61] Voigt C , Marushchak ME, Lamprecht RE et al. Increased nitrous oxide emissions from Arctic peatlands after permafrost thaw. Proc Nat Acad Sci USA. 2017;114:6238–43. 28559346 10.1073/pnas.1702902114 PMC5474798

[bib62] Walker DA , Raynolds MK, Daniëls FJA et al. The circumpolar Arctic vegetation map. J Veg Sci. 2005;16:267–82.

[bib63] Wang H , Teng C, Li H et al. Microbial community shifts trigger loss of orthophosphate in wetland soils subjected to experimental warming. Plant Soil. 2018;424:351–65.

[bib64] Ward SE , Ostle NJ, Oakley S et al. Warming effects on greenhouse gas fluxes in peatlands are modulated by vegetation composition. Ecol Lett. 2013;16:1285–93. 23953244 10.1111/ele.12167

[bib65] Wookey PA , Parsons NA, Welker JM et al. Comparative responses of phenology and reproductive development to simulated environmental change in subArctic and high Arctic. OIKOS. 1993;67:490–502.

[bib66] Xiong J , Chu H, Sun H et al. Divergent responses of soil fungi functional groups to short-term warming. Microb Ecol. 2014;68::708–15. 24553914 10.1007/s00248-014-0385-6

[bib67] Xu X , Liu Y, Singh BP et al. NosZ clade II rather than clade I determine *in situ* N_2_O emissions with different fertiliser types under simulated climate change and its legacy. Soil Biol Biochem. 2020a;150::10794.

[bib68] Xu X , Zhang M, Xiong Y et al. The influence of soil temperature, methanogens and methanotrophs on methane emissions from cold waterlogged paddy fields. J Environ Manage. 2020b;264:110421. 32217313 10.1016/j.jenvman.2020.110421

[bib69] Yue H , Wang M, Wang S et al. The microbe-mediated mechanisms affecting topsoil carbon stock in Tibetan grasslands. ISME J. 2015;9:2012–20. 25689025 10.1038/ismej.2015.19 PMC4542033

[bib70] Zhang W , Kang X, Kang E et al. Soil water content, carbon, and nitrogen determine the abundances of methanogens, methanotrophs, and methane emission in the Zoige alpine wetland. J Soils Sediments. 2022;22:470–81.

[bib71] Zhang Z , Wang H, Zhou J et al. Redox potential and microbial functional gene diversity in wetland sediments under simulated warming conditions: implications for phosphorus mobilisation. Hydrobiologia. 2015;743:221–35.

