# Supplementary material for: Vegetation type, not the legacy of warming, modifies the response of microbial functional genes and greenhouse gas fluxes to drought in Oro-Arctic and alpine regions
Source: FEMS Microbiol Ecol. 2023 Nov 10;99(12):fiad145. doi: 10.1093/femsec/fiad145 (PMC10673709; doi:10.1093/femsec/fiad145)
Supplement: fiad145_Supplemental_Files [file fiad145_supplemental_files.zip › Supplementary data code 2 gene abundance.docx]

library(knitr)

library(nlme)

library(lattice)

library(multcomp)

library(emmeans)

library(rlang)

library(ggplot2)

library(scales)

library(ggthemes)

library(cowplot)

library(stringr)

library(patchwork)

library(lme4)

qPCR.data.for.R.day.0.and.56 <- read.delim("C:/Users/fryel/OneDrive - Edge Hill University/Resistance experiment 2/DNA stuff/qPCR/qPCR data for R day 0 and 56.txt")

View(qPCR.data.for.R.day.0.and.56)

attach(qPCR.data.for.R.day.0.and.56)

fieldrep<-factor(Fieldrep)

exptrep<-factor(Exptrep)

#########################################################################################

#########################################################################################

#Gene ANOVA

m1a<-lmer(log(pmoaratio)~(Timepoint+Plot+Drought+Warming)^3+(1|fieldrep),na.action=na.omit)

m1b<-lm(log(pmoaratio)~(Timepoint+Plot+Drought+Warming)^3,na.action=na.omit)

anova(m1a,m1b)

r.squaredGLMM(m1b)

groups<-interaction(Plot, Warming,Timepoint)

model_posthoc<-with(qPCR.data.for.R.day.0.and.56, glm(log(pmoaratio) ~ groups, family=gaussian))

summary(glht(model_posthoc,linfct=mcp(groups="Tukey")))

amod <- aov(log(pmoaratio) ~groups)

tukey<-TukeyHSD(amod)

model_means <- emmeans(object = amod,

specs = ~ groups)

model_means_cld <- cld(object = model_means,

adjust = "Tukey",

Letters = letters,

alpha = 0.05)

model_means_cld

###################################

m1a<-lmer(log(X16S)~(Timepoint+Plot+Drought+Warming)^3+(1|fieldrep),na.action=na.omit)

m1b<-lm(log(X16S)~(Timepoint+Plot+Drought+Warming)^3,na.action=na.omit)

anova(m1a,m1b)

anova(m1a)

r.squaredGLMM(m1a)

m1a<-lme(log(X16S)~(Timepoint+Plot+Drought+Warming)^3,random=~1|fieldrep,na.action=na.omit)

anova(m1a)

groups<-interaction(Plot, Timepoint)

model_posthoc<-with(qPCR.data.for.R.day.0.and.56, glm(log(X16S) ~ groups, family=gaussian))

summary(glht(model_posthoc,linfct=mcp(groups="Tukey")))

amod <- aov(log(X16S) ~groups)

tukey<-TukeyHSD(amod)

model_means <- emmeans(object = amod,

specs = ~ groups)

model_means_cld <- cld(object = model_means,

adjust = "Tukey",

Letters = letters,

alpha = 0.05)

model_means_cld

m1a<-lmer(log(ITS)~(Timepoint+Plot+Drought+Warming)^3+(1|fieldrep),na.action=na.omit)

m1b<-lm(log(ITS)~(Timepoint+Plot+Drought+Warming)^3,na.action=na.omit)

anova(m1a,m1b)

anova(m1b)

r.squaredGLMM(m1b)

groups<-interaction(Plot, Timepoint,Warming)

model_posthoc<-with(qPCR.data.for.R.day.0.and.56, glm(log(ITS) ~ groups, family=gaussian))

summary(glht(model_posthoc,linfct=mcp(groups="Tukey")))

amod <- aov(log(ITS) ~groups)

tukey<-TukeyHSD(amod)

model_means <- emmeans(object = amod,

specs = ~ groups)

model_means_cld <- cld(object = model_means,

adjust = "Tukey",

Letters = letters,

alpha = 0.05)

model_means_cld

m1a<-lmer(log(nosratio)~(Timepoint+Plot+Drought+Warming)^3+(1|fieldrep),na.action=na.omit)

m1b<-lm(log(nosratio)~(Timepoint+Plot+Drought+Warming)^3,na.action=na.omit)

anova(m1a,m1b)

m1a<-lme(log(nosratio)~(Timepoint+Plot+Drought+Warming)^3,random=~1|fieldrep,na.action=na.omit)

anova(m1a)

r.squaredGLMM(m1a)

groups<-interaction(Plot, Timepoint)

model_posthoc<-with(qPCR.data.for.R.day.0.and.56, glm(log(nosratio) ~ groups, family=gaussian))

summary(glht(model_posthoc,linfct=mcp(groups="Tukey")))

amod <- aov(log(nosratio) ~groups)

tukey<-TukeyHSD(amod)

model_means <- emmeans(object = amod,

specs = ~ groups)

model_means_cld <- cld(object = model_means,

adjust = "Tukey",

Letters = letters,

alpha = 0.05)

model_means_cld

m1a<-lmer(log(acdsratio)~(Timepoint+Plot+Drought+Warming)^3+(1|fieldrep),na.action=na.omit)

m1b<-lm(log(acdsratio)~(Timepoint+Plot+Drought+Warming)^3,na.action=na.omit)

anova(m1a,m1b)

anova(m1b)

r.squaredGLMM(m1b)

groups<-interaction(Plot, Timepoint,Warming)

model_posthoc<-with(qPCR.data.for.R.day.0.and.56, glm(log(acdsratio) ~ groups, family=gaussian))

summary(glht(model_posthoc,linfct=mcp(groups="Tukey")))

amod <- aov(log(acdsratio) ~groups)

tukey<-TukeyHSD(amod)

model_means <- emmeans(object = amod,

specs = ~ groups)

model_means_cld <- cld(object = model_means,

adjust = "Tukey",

Letters = letters,

alpha = 0.05)

model_means_cld

########################################################################

#

## CODE FOR THE VERY SMALL NUMBER NOTATION

fancy_scientific <- function(l) {

# turn in to character string in scientific notation

l <- format(l, scientific = TRUE)

# quote the part before the exponent to keep all the digits

l <- gsub("^(.*)e", "'\\1'e", l)

# turn the 'e+' into plotmath format

l <- gsub("e", "%*%10^", l)

# return this as an expression

parse(text=l)

}

#

position_jitterdodge(

jitter.width = NULL,

jitter.height = 0,

dodge.width = 0.75,

seed = NA

)

pd <- position_dodge(0.1)

qPCR.data.for.R.day.0.and.56$Plot<- factor(qPCR.data.for.R.day.0.and.56$Plot,levels = c("2Cassiope", "3Eriophorum","1Ranunculus","4Saxifraga"))

levels(qPCR.data.for.R.day.0.and.56$Plot) <- list('Sweden Wet meadow'="1Ranunculus",'Sweden Dry heath'="2Cassiope", 'Sweden Tussock tundra'="3Eriophorum",'Switzerland Alpine'="4Saxifraga")

qPCR.data.for.R.day.0.and.56$Plot

qPCR.data.for.R.day.0.and.56$Drought<- factor(qPCR.data.for.R.day.0.and.56$Drought,levels = c("Wet", "Dry"))

levels(qPCR.data.for.R.day.0.and.56$Drought) <- list('Control'="Wet", 'Drought'="Dry")

qPCR.data.for.R.day.0.and.56$Drought

qPCR.data.for.R.day.0.and.56$Warming<- factor(qPCR.data.for.R.day.0.and.56$Warming,levels = c("C", "W"))

levels(qPCR.data.for.R.day.0.and.56$Warming) <- list('Ambient'="C", 'Warmed'="W")

qPCR.data.for.R.day.0.and.56$Warming

time<-factor(Timepoint)

pmmo<-ggplot(qPCR.data.for.R.day.0.and.56, aes(y=pmoaratio, x=Plot, linetype=Warming,colour=Drought)) +

theme_bw(base_size=20) +

geom_boxplot( size = .5) +

#geom_point(size=.5, position = position_jitterdodge()) +

scale_y_continuous(trans="log10",labels=fancy_scientific)+

scale_x_discrete(labels = function(x) str_wrap(x, width = 10))+

scale_color_manual(values = c("Control" = "darkblue", "Drought"="orange")) +

ylab(expression("pmoA:16S gene abundance")) +

xlab("") +

theme(text = element_text(size=13),axis.text = element_text(size =11),axis.text.x = element_text(angle = 45, vjust = 0.5))+

theme(plot.margin = unit(c(0,0,0,0), "cm")) +

theme(panel.grid.major = element_blank(), panel.grid.minor = element_blank())+

theme(legend.position="bottom",legend.title = element_blank(),legend.direction = "vertical")+

theme(axis.line.x = element_line(color="black", size = 0.5),

axis.line.y = element_line(color="black", size =0.5))+

facet_wrap(time)

pmmo

fung<-ggplot(qPCR.data.for.R.day.0.and.56, aes(y=ITS, x=Plot,linetype=Warming, colour=Drought)) +

theme_bw(base_size=20) +

geom_boxplot( size = .5) +

#geom_point(size=.5, position = position_jitterdodge()) +

scale_y_continuous(trans="log10",labels=fancy_scientific)+

scale_x_discrete(labels = function(x) str_wrap(x, width = 10))+

scale_color_manual(values = c("Control" = "darkblue", "Drought"="orange")) +

ylab(expression("ITS gene abudance")) +

xlab("") +

theme(text = element_text(size=13),axis.text = element_text(size =11),axis.text.x = element_text(angle = 45, vjust = 0.5))+

theme(plot.margin = unit(c(0,0,0,0), "cm")) +

theme(panel.grid.major = element_blank(), panel.grid.minor = element_blank())+

theme(legend.position="bottom",legend.title = element_blank(),legend.direction = "vertical")+

theme(axis.line.x = element_line(color="black", size = 0.5),

axis.line.y = element_line(color="black", size =0.5))+

facet_wrap(time)

fung

stress<-ggplot(qPCR.data.for.R.day.0.and.56, aes(y=acdsratio, x=Plot,linetype=Warming, colour=Drought)) +

theme_bw(base_size=20) +

geom_boxplot( size = .5) +

#geom_point(size=.5, position = position_jitterdodge()) +

scale_y_continuous(trans="log10",labels=fancy_scientific)+

scale_x_discrete(labels = function(x) str_wrap(x, width = 10))+

scale_color_manual(values = c("Control" = "darkblue", "Drought"="orange")) +

ylab(expression("acdS:16S gene abudance")) +

xlab("") +

theme(text = element_text(size=13),axis.text = element_text(size =11),axis.text.x = element_text(angle = 45, vjust = 0.5))+

theme(plot.margin = unit(c(0,0,0,0), "cm")) +

theme(panel.grid.major = element_blank(), panel.grid.minor = element_blank())+

theme(legend.position="bottom",legend.title = element_blank(),legend.direction = "vertical")+

theme(axis.line.x = element_line(color="black", size = 0.5),

axis.line.y = element_line(color="black", size =0.5))+

facet_wrap(time)

stress

bact<-ggplot(qPCR.data.for.R.day.0.and.56, aes(y=X16S, x=Plot,linetype=Warming,colour=Drought)) +

theme_bw(base_size=20) +

geom_boxplot( size = .5) +

#geom_point(size=.5) +

scale_y_continuous(trans="log10",labels=fancy_scientific)+

scale_x_discrete(labels = function(x) str_wrap(x, width = 10))+

scale_color_manual(values = c("Control" = "darkblue", "Drought"="orange")) +

ylab(expression("16S gene abudance")) +

xlab("") +

theme(text = element_text(size=13),axis.text = element_text(size =11),axis.text.x = element_text(angle = 45, vjust = 0.5))+

theme(plot.margin = unit(c(0,0,0,0), "cm")) +

theme(panel.grid.major = element_blank(), panel.grid.minor = element_blank())+

theme(legend.position="bottom",legend.title = element_blank(),legend.direction = "vertical")+

theme(axis.line.x = element_line(color="black", size = 0.5),

axis.line.y = element_line(color="black", size =0.5))+

facet_wrap(time)

bact

nit<-ggplot(qPCR.data.for.R.day.0.and.56, aes(y=nosratio, x=Plot,linetype=Warming,colour=Drought)) +

theme_bw(base_size=20) +

geom_boxplot( size = .5) +

# geom_point(size=.5) +

scale_y_continuous(trans="log10",labels=fancy_scientific)+

scale_x_discrete(labels = function(x) str_wrap(x, width = 10))+

scale_color_manual(values = c("Control" = "darkblue", "Drought"="orange")) +

ylab(expression("nosZII:16S gene abudance")) +

xlab("") +

theme(text = element_text(size=13),axis.text = element_text(size =11),axis.text.x = element_text(angle = 45, vjust = 0.5))+

theme(plot.margin = unit(c(0,0,0,0), "cm")) +

theme(panel.grid.major = element_blank(), panel.grid.minor = element_blank())+

theme(legend.position="bottom",legend.title = element_blank(),legend.direction = "vertical")+

theme(axis.line.x = element_line(color="black", size = 0.5),

axis.line.y = element_line(color="black", size =0.5))+

facet_wrap(time)

nit

patchwork<-bact / fung / pmmo / nit /stress+guide_area() +

plot_layout(guides = 'collect')

patchwork# export as 750x2500
