# Supplementary material for: Vegetation type, not the legacy of warming, modifies the response of microbial functional genes and greenhouse gas fluxes to drought in Oro-Arctic and alpine regions
Source: FEMS Microbiol Ecol. 2023 Nov 10;99(12):fiad145. doi: 10.1093/femsec/fiad145 (PMC10673709; doi:10.1093/femsec/fiad145)
Supplement: fiad145_Supplemental_Files [file fiad145_supplemental_files.zip › Supplementary data.docx]

SUPPLEMENTARY DATA

Figure S1: Experiment schematic showing the field treatments and the drought treatment and recovery period in the growth cabinet.


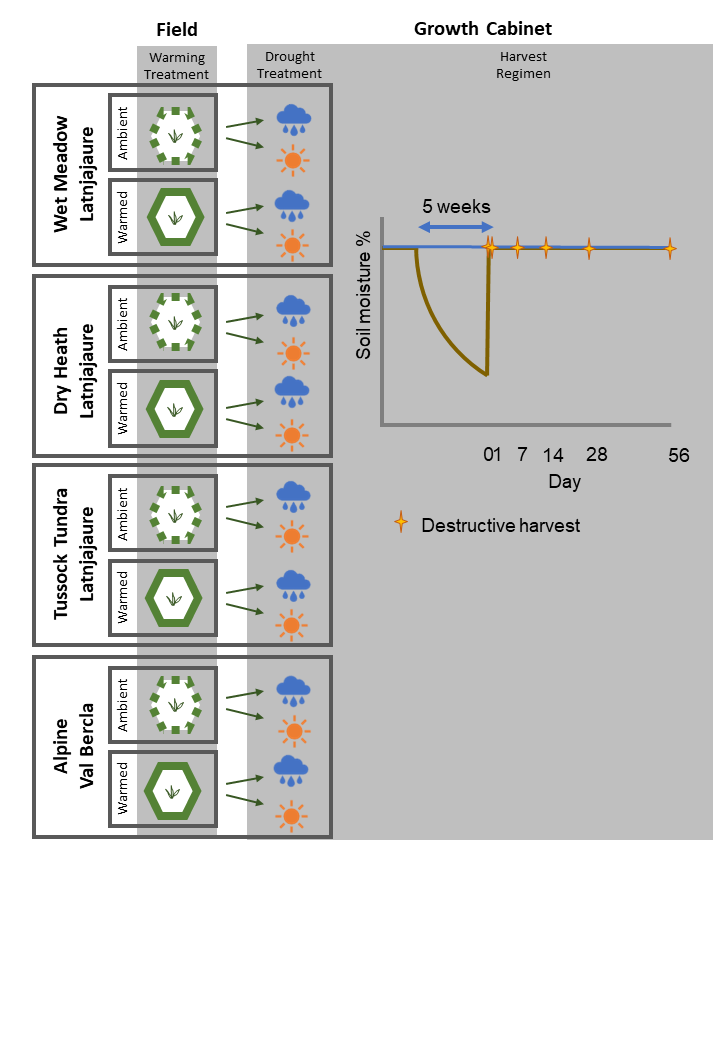


Figure S2: Soil moisture content at the start of the study. Error bars are standard error of the mean.


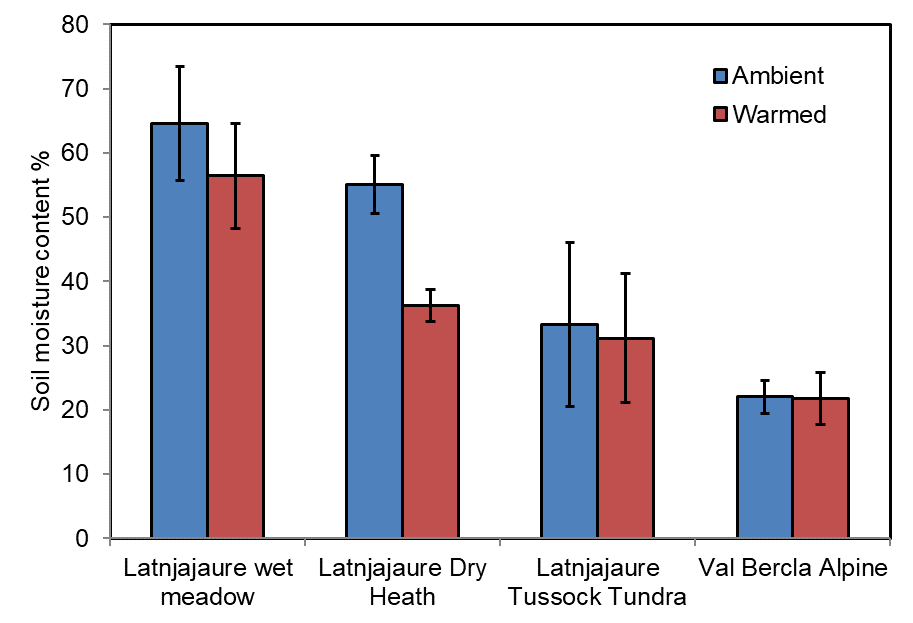


Table S1: Primers used in real-time PCR, with cycling conditions (Martins et al. 2017).

| Gene | Primer sequence | Warmup | Cycle | Number of cycles |
| --- | --- | --- | --- | --- |
| 16S | Eub338: ACT CCT ACG GGA GGC AGC AG  Eub518: ATT ACC GCG GCT GCT GG | 95˚C for 3 minutes | 95˚C for 15s  53˚C for 15s  72˚C for 15s  Dissociation curve:  95˚C for 30s  65˚C for 60s  97˚C continuously | 40 |
| ITS | ITS1f: TCC GTA GGT GAA CCT GCG G  5.8s: CGC TGC GTT CTT CAT CG | 95˚C for 3 minutes | 95˚C for 15s  53˚C for 15s  72˚C for 15s  Dissociation curve:  95˚C for 30s  65˚C for 60s  97˚C continuously | 40 |
| acdS | acdSF: GGC AAC AAG CGC AAG CT  acdSR: CTG CAC AGA CGC ACT TCA | 95˚C for 10 minutes | 95˚C for 15s  67˚C for 10s  72˚C for 30s  Dissociation curve:  95˚C for 30s  65˚C for 60s  97˚C continuously | 50 |
| nosZ | nosZ2-F: CGC ACG GCA AAA GGT GT  nosZ2-R: ATG CGC TGG CAG AA | 95˚C for 3 minutes | 95˚C for 15s  60˚C for 10s  72˚C for 20s  Dissociation curve:  95˚C for 30s  65˚C for 60s  97˚C continuously | 40 |
| pmoA | pmo189-F: GGN GAC TGG GAC TTC TGG  pmo650-R: ACG TCC TTA CG AAG GT | 95˚C for 3 minutes | 95˚C for 15s  53˚C for 10s  72˚C for 30s  Dissociation curve:  95˚C for 30s  65˚C for 60s  97˚C continuously | 40 |

Table S2: Statistical output of linear mixed effects models, looking at relationships between functional gene abundance and greenhouse gas emissions. Results are only presented graphically when a gene is significantly related to the treatments to impact greenhouse gas fluxes. Results significant at the p<0.05 level are highlighted in bold. R^2^c refers to conditional R^2^ model fit (fixed and random effects), while R^2^m is marginal (fixed effects only). Where likelihood ratio deletion tests recommend removal of random effects, these two values will be the same.

| Gene | Variables | df | Carbon dioxide | | Methane | | Nitrous oxide | | | |
| --- | --- | --- | --- | --- | --- | --- | --- | --- | --- | --- |
|  |  |  |  |  |  |  |  | |  | |
|  |  |  | F | p | F | p | F | | p | |
| 16S (bacteria) | intercept | 1,67 | 257.28 | <0.001 | 0.11 | 0.744 | 38.85 | | <.0001 | |
|  | Vegetation (V) | 3,67 | **4.35** | **0.007** | 0.76 | 0.518 | 1.01 | | 0.392 | |
|  | Drought (D) | 1,67 | **9.04** | **0.004** | 0.01 | 0.932 | 1.18 | | 0.282 | |
|  | Warming (W) | 1,67 | 0.09 | 0.762 | 0.75 | 0.388 | 0.03 | | 0.858 | |
|  | GENE | 1,67 | 0.01 | 0.907 | 0.40 | 0.531 | **7.13** | | **0.010** | |
|  | V x D | 3,67 | **4.20** | **0.009** | 0.86 | 0.465 | 0.32 | | 0.810 | |
|  | V x W | 3,67 | 0.46 | 0.715 | 0.61 | 0.612 | **3.48** | | **0.021** | |
|  | V x GENE | 3,67 | 0.31 | 0.818 | 0.75 | 0.527 | 2.27 | | 0.088 | |
|  | D x W | 1,67 | 2.53 | 0.117 | 1.58 | 0.214 | 1.19 | | 0.279 | |
|  | D x GENE | 1,67 | **7.72** | **0.007** | 0.05 | 0.825 | 3.69 | | 0.059 | |
|  | W x GENE | 1,67 | 0.00 | 0.973 | 0.04 | 0.847 | 0.02 | | 0.885 | |
|  | V x D x W | 3,67 | 0.91 | 0.442 | 0.54 | 0.660 | 0.66 | | 0.582 | |
|  | V x D x GENE | 3,67 | 0.46 | 0.710 | 1.17 | 0.329 | 0.44 | | 0.726 | |
|  | V x W x GENE | 3,67 | 0.61 | 0.610 | 2.41 | 0.074 | 1.38 | | 0.256 | |
|  | D x W x GENE | 1,67 | **4.69** | **0.033** | 1.55 | 0.218 | 1.00 | | 0.321 | |
|  |  |  | R^2^c | R^2^m | R^2^c | R^2^m | R^2^c | | R^2^m | |
|  |  |  | 0.379 | 0.379 | 0.213 | 0.213 | 0.508 | | 0.302 | |
| ITS (fungi) | intercept | 1,67 | 252.97 | <.0001 | 0.11 | 0.739 | 35.68 | | <.0001 | |
|  | Vegetation (V) | 3,67 | 4.28 | 0.008 | 0.80 | 0.501 | 0.93 | | 0.430 | |
|  | Drought (D) | 1,67 | 8.89 | 0.004 | 0.01 | 0.930 | 1.08 | | 0.302 | |
|  | Warming (W) | 1,67 | 0.09 | 0.764 | 0.79 | 0.379 | 0.03 | | 0.864 | |
|  | GENE | 1,67 | 2.83 | 0.097 | 0.08 | 0.775 | 1.29 | | 0.261 | |
|  | V x D | 3,67 | **4.22** | **0.009** | 0.92 | 0.438 | 0.37 | | 0.777 | |
|  | V x W | 3,67 | 0.30 | 0.827 | 0.61 | 0.611 | **3.22** | | **0.028** | |
|  | V x GENE | 3,67 | 0.50 | 0.683 | 0.65 | 0.585 | 0.88 | | 0.459 | |
|  | D x W | 1,67 | 2.61 | 0.111 | 2.07 | 0.155 | 1.90 | | 0.172 | |
|  | D x GENE | 1,67 | 1.40 | 0.241 | 0.01 | 0.935 | 3.03 | | 0.086 | |
|  | W x GENE | 1,67 | 0.17 | 0.681 | 1.33 | 0.252 | 0.16 | | 0.691 | |
|  | V x D x W | 3,67 | 1.015 | 0.392 | 0.74 | 0.534 | 0.79 | | 0.503 | |
|  | V x D x GENE | 3,67 | 1.15 | 0.335 | 1.51 | 0.221 | 0.93 | | 0.433 | |
|  | V x W x GENE | 3,67 | 0.80 | 0.497 | 2.56 | 0.062 | 1.71 | | 0.174 | |
|  | D x W x GENE | 1,67 | 3.10 | 0.083 | 1.91 | 0.172 | 0.00 | | 0.996 | |
|  |  |  | R^2^c | R^2^m | R^2^c | R^2^m | R^2^c | | R^2^m | |
|  |  |  | 0.368 | 0.368 | 0.238 | 0.238 | 0.261 | | 0.261 | |
| *acdS*:16S | intercept | 1,67 | 241.92 | <.0001 | NA |  | NA | |  | |
| (stress gene) | Vegetation (V) | 3,67 | **4.09** | **0.010** |  |  |  | |  | |
|  | Drought (D) | 1,67 | **8.50** | **0.005** |  |  |  | |  | |
|  | Warming (W) | 1,67 | 0.09 | 0.769 |  |  |  | |  | |
|  | GENE | 1,67 | 0.03 | 0.857 |  |  |  | |  | |
|  | V x D | 3,67 | **4.00** | **0.011** |  |  |  | |  | |
|  | V x W | 3,67 | 0.51 | 0.678 |  |  |  | |  | |
|  | V x GENE | 3,67 | 2.29 | 0.086 |  |  |  | |  | |
|  | D x W | 1,67 | 2.02 | 0.160 |  |  |  | |  | |
|  | D x GENE | 1,67 | 0.09 | 0.772 |  |  |  | |  | |
|  | W x GENE | 1,67 | 2.90 | 0.093 |  |  |  | |  | |
|  | V x D x W | 3,67 | 0.51 | 0.674 |  |  |  | |  | |
|  | V x D x GENE | 3,67 | 0.47 | 0.702 |  |  |  | |  | |
|  | V x W x GENE | 3,67 | 0.34 | 0.799 |  |  |  | |  | |
|  | D x W x GENE | 1,67 | 0.24 | 0.626 |  |  |  | |  | |
|  |  |  | R^2^c | R^2^m |  |  |  | |  | |
|  |  |  | 0.347 | 0.347 |  |  |  | |  | |
| *pmoA*:16S | intercept | 1,67 | 292.07 | <0.001 | 0.12 | 0.733 | | NA | | |
| (methanotroph) | Vegetation (V) | 3,67 | **4.94** | **0.004** | 0.83 | 0.480 |  | |  | |
|  | Drought (D) | 1,67 | **10.26** | **0.002** | 0.01 | 0.929 |  | |  | |
|  | Warming (W) | 1,67 | 0.11 | 0.747 | 0.82 | 0.368 |  | |  | |
|  | GENE | 1,67 | 0.55 | 0.459 | 0.26 | 0.611 |  | |  | |
|  | V x D | 3,67 | **4.89** | **0.004** | 0.91 | 0.441 |  | |  | |
|  | V x W | 3,67 | 0.64 | 0.595 | 0.63 | 0.601 |  | |  | |
|  | V x GENE | 3,67 | **4.48** | **0.006** | **3.11** | **0.032** |  | |  | |
|  | D x W | 1,67 | 0.87 | 0.354 | 0.80 | 0.375 |  | |  | |
|  | D x GENE | 1,67 | 0.05 | 0.820 | 1.39 | 0.243 |  | |  | |
|  | W x GENE | 1,67 | 1.84 | 0.179 | 0.28 | 0.596 |  | |  | |
|  | V x D x W | 3,67 | 0.76 | 0.521 | 1.25 | 0.299 |  | |  | |
|  | V x D x GENE | 3,67 | **3.13** | **0.031** | 2.36 | 0.080 |  | |  | |
|  | V x W x GENE | 3,67 | 1.31 | 0.278 | 0.99 | 0.402 |  | |  | |
|  | D x W x GENE | 1,67 | 0.71 | 0.402 | 0.31 | 0.578 |  | |  | |
|  |  |  | R^2^c | R^2^m | R^2^c | R^2^m |  | |  | |
|  |  |  | 0.441 | 0.441 | 0.264 | 0.264 |  | |  | |
| *nosZ II*:16S | intercept | 1,40 | NA | | NA | | | 20.86 | | <.001 |
| (nitrous oxide breakdown) | Vegetation (V) | 3,40 |  |  |  |  | 0.54 | | 0.653 | |
|  | Drought (D) | 1,23 |  |  |  |  | 1.88 | | 0.182 | |
|  | Warming (W) | 1,40 |  |  |  |  | 0.01 | | 0.906 | |
|  | GENE | 1,23 |  |  |  |  | 3.11 | | 0.089 | |
|  | V x D | 3,23 |  |  |  |  | 0.66 | | 0.585 | |
|  | V x W | 3,40 |  |  |  |  | 1.75 | | 0.173 | |
|  | V x GENE | 3,23 |  |  |  |  | 1.04 | | 0.393 | |
|  | D x W | 1,23 |  |  |  |  | 2.28 | | 0.143 | |
|  | D x GENE | 1,23 |  |  |  |  | 0.04 | | 0.841 | |
|  | W x GENE | 1,23 |  |  |  |  | 0.10 | | 0.753 | |
|  | V x D x W | 3,23 |  |  |  |  | 1.48 | | 0.243 | |
|  | V x D x GENE | 3,23 |  |  |  |  | 0.61 | | 0.614 | |
|  | V x W x GENE | 3,23 |  |  |  |  | 1.13 | | 0.357 | |
|  | D x W x GENE | 1,23 |  |  |  |  | 0.75 | | 0.396 | |
|  |  |  |  |  |  |  | R^2^c | | R^2^m | |
|  |  |  |  |  |  |  | 0.536 | | 0.209 | |
