# Supplementary material for: Vegetation type, not the legacy of warming, modifies the response of microbial functional genes and greenhouse gas fluxes to drought in Oro-Arctic and alpine regions
Source: FEMS Microbiol Ecol. 2023 Nov 10;99(12):fiad145. doi: 10.1093/femsec/fiad145 (PMC10673709; doi:10.1093/femsec/fiad145)
Supplement: fiad145_Supplemental_Files [file fiad145_supplemental_files.zip › Supplementarydata code 3 gene abundance vs GHG.docx]

library(knitr)

library(nlme)

library(lattice)

library(multcomp)

library(emmeans)

library(rlang)

library(cowplot)

library(stringr)

library(patchwork)

library(ggplot2)

library(scales)

library(ggthemes)

library(patchwork)

library(gridExtra)

library(ggExtra)

library(MuMIn)

install.packages("piecewiseSEM")

library(piecewiseSEM)

library(lme4)

library(multcompView)

qPCR.data.for.R.day.0.and.56 <- read.delim("C:/Users/fryel/OneDrive - Edge Hill University/Resistance experiment 2/DNA stuff/qPCR/qPCR data for R day 0 and 56.txt")

View(qPCR.data.for.R.day.0.and.56)

attach(qPCR.data.for.R.day.0.and.56)

##########################################################################################

##########################################################################################

#Treatment effects on ghgs

#CO2

m1a<-lmer(CO2~(Timepoint+Plot+Drought+Warming)^3+(1|fieldrep),na.action=na.omit)

m1b<-lm(CO2~(Timepoint+Plot+Drought+Warming)^3,na.action=na.omit)

anova(m1a,m1b)

anova(m1b)

r.squaredGLMM(m1b)

groups<-interaction(Timepoint,Drought, Plot)

model_posthoc<-with(qPCR.data.for.R.day.0.and.56, glm(CO2 ~ groups, family=gaussian))

summary(glht(model_posthoc,linfct=mcp(groups="Tukey")))

amod <- aov(CO2 ~groups)

tukey<-TukeyHSD(amod)

model_means <- emmeans(object = amod,

specs = ~ groups)

model_means_cld <- cld(object = model_means,

adjust = "sidak",

Letters = letters,

alpha = 0.05)

model_means_cld

#CH4

m1a<-lmer(CH4~(Timepoint+Plot+Drought+Warming)^3+(1|fieldrep),na.action=na.omit)

m1b<-lm(CH4~(Timepoint+Plot+Drought+Warming)^3,na.action=na.omit)

anova(m1a,m1b)

anova(m1b)

r.squaredGLMM(m1b)

groups<-interaction(Timepoint,Warming, Plot)

model_posthoc<-with(qPCR.data.for.R.day.0.and.56, glm(CH4 ~ groups, family=gaussian))

summary(glht(model_posthoc,linfct=mcp(groups="Tukey")))

amod <- aov(CH4 ~groups)

tukey<-TukeyHSD(amod)

model_means <- emmeans(object = amod,

specs = ~ groups)

model_means_cld <- cld(object = model_means,

adjust = "sidak",

Letters = letters,

alpha = 0.05)

model_means_cld

#N2O

fieldrep<-factor(Fieldrep)

exptrep<-factor(Exptrep)

m1a<-lmer(N2O~(Timepoint+Plot+Drought+Warming)^3+(1|fieldrep),na.action=na.omit)

m1b<-lm(N2O~(Timepoint+Plot+Drought+Warming)^3,na.action=na.omit)

anova(m1a,m1b)

anova(m1b)

r.squaredGLMM(m1b)

groups<-interaction(Timepoint,Drought, Warming)

model_posthoc<-with(qPCR.data.for.R.day.0.and.56, glm(N2O ~ groups, family=gaussian))

summary(glht(model_posthoc,linfct=mcp(groups="Tukey")))

amod <- aov(N2O ~groups)

tukey<-TukeyHSD(amod)

model_means <- emmeans(object = amod,

specs = ~ groups)

model_means_cld <- cld(object = model_means,

adjust = "sidak",

Letters = letters,

alpha = 0.05)

model_means_cld

#######################################################################################

#######################################################################################

#Greenhouse gas

##

time<-factor(Timepoint)

remove_outliers <- function(x, na.rm = TRUE, ...) {

qnt <- quantile(x, probs=c(.25, .75), na.rm = na.rm, ...)

H <- 1.5 * IQR(x, na.rm = na.rm)

y <- x

y[x < (qnt[1] - H)] <- NA

y[x > (qnt[2] + H)] <- NA

y

}

qPCR.data.for.R.day.0.and.56$Plot<- factor(qPCR.data.for.R.day.0.and.56$Plot,levels = c("2Cassiope", "3Eriophorum","1Ranunculus","4Saxifraga"))

levels(qPCR.data.for.R.day.0.and.56$Plot) <- list('Sweden Wet meadow'="1Ranunculus",'Sweden Dry heath'="2Cassiope", 'Sweden Tussock tundra'="3Eriophorum",'Switzerland Alpine'="4Saxifraga")

qPCR.data.for.R.day.0.and.56$Plot

qPCR.data.for.R.day.0.and.56$Drought<- factor(qPCR.data.for.R.day.0.and.56$Drought,levels = c("Wet", "Dry"))

levels(qPCR.data.for.R.day.0.and.56$Drought) <- list('Control'="Wet", 'Drought'="Dry")

qPCR.data.for.R.day.0.and.56$Drought

qPCR.data.for.R.day.0.and.56$Warming<- factor(qPCR.data.for.R.day.0.and.56$Warming,levels = c("C", "W"))

levels(qPCR.data.for.R.day.0.and.56$Warming) <- list('Ambient'="C", 'Warmed'="W")

qPCR.data.for.R.day.0.and.56$Warming

carb<-ggplot(qPCR.data.for.R.day.0.and.56, aes(y=CO2, x=Plot,linetype=Warming, colour=Drought)) +

theme_bw(base_size=20) +

geom_boxplot( size = .5) +

#geom_point(size=.5, aes(color=Drought), position = position_jitterdodge()) +

scale_y_continuous(trans="log10",labels=fancy_scientific)+

scale_x_discrete(labels = function(x) str_wrap(x, width = 10))+

scale_color_manual(values = c("Control" = "darkblue", "Drought"="orange")) +

ylab(expression("Carbon dioxide")) +

xlab("") +

theme(text = element_text(size=13),axis.text = element_text(size =11),axis.text.x = element_text(angle = 45, vjust = 0.5))+

theme(plot.margin = unit(c(0,0,0,0), "cm")) +

theme(panel.grid.major = element_blank(), panel.grid.minor = element_blank())+

theme(legend.position="bottom",legend.title = element_blank(),legend.direction = "vertical")+

theme(axis.line.x = element_line(color="black", size = 0.5),

axis.line.y = element_line(color="black", size =0.5))+

facet_wrap(time)

carb

meth<-ggplot(qPCR.data.for.R.day.0.and.56, aes(y=CH4, x=Plot,linetype=Warming, colour=Drought)) +

theme_bw(base_size=20) +

geom_boxplot( size = .5) +

#geom_point(size=.5, position = position_jitterdodge()) +

scale_y_continuous(trans="log10",labels=fancy_scientific)+

scale_x_discrete(labels = function(x) str_wrap(x, width = 10))+

scale_color_manual(values = c("Control" = "darkblue", "Drought"="orange")) +

ylab(expression("Methane")) +

xlab("") +

theme(text = element_text(size=13),axis.text = element_text(size =11),axis.text.x = element_text(angle = 45, vjust = 0.5))+

theme(plot.margin = unit(c(0,0,0,0), "cm")) +

theme(panel.grid.major = element_blank(), panel.grid.minor = element_blank())+

theme(legend.position="bottom",legend.title = element_blank(),legend.direction = "vertical")+

theme(axis.line.x = element_line(color="black", size = 0.5),

axis.line.y = element_line(color="black", size =0.5))+

facet_wrap(time)

meth

nit<-ggplot(qPCR.data.for.R.day.0.and.56, aes(y=N2O, x=Plot,linetype=Warming, color=Drought)) +

theme_bw(base_size=20) +

geom_boxplot(aes(color = Drought), size = .5) +

#geom_point(size=.5, aes(color=Drought), position = position_jitterdodge()) +

scale_y_continuous(trans="log10",labels=fancy_scientific)+

scale_x_discrete(labels = function(x) str_wrap(x, width = 10))+

scale_color_manual(values = c("Control" = "darkblue", "Drought"="orange")) +

ylab(expression("Nitrous oxide")) +

xlab("") +

theme(text = element_text(size=13),axis.text = element_text(size =11),axis.text.x = element_text(angle = 45, vjust = 0.5))+

theme(plot.margin = unit(c(0,0,0,0), "cm")) +

theme(panel.grid.major = element_blank(), panel.grid.minor = element_blank())+

theme(legend.position="bottom",legend.title = element_blank(),legend.direction = "vertical")+

theme(axis.line.x = element_line(color="black", size = 0.5),

axis.line.y = element_line(color="black", size =0.5))+

facet_wrap(time)

nit

carb/meth/nit+guide_area() +

plot_layout(guides = 'collect')# export 750 width, 1500 height

####

#####################################################################################

### GENES VS GHGs - Day 56 only.

day56only<-qPCR.data.for.R.day.0.and.56[c(97:192),]

fieldrep<-factor(day56only$Fieldrep)

#########################################################################################

#########################################################################################

## N2O

#

#anova - nosZ

dotchart(log(day56only$nosratio))

dotchart(day56only$N2O)

m1a.nosz<-lmer(N2O~(Plot+Drought+Warming+log(nosratio))^3+(1|fieldrep),na.action=na.omit,day56only)

m1b.nosz<-lm(N2O~(Plot+Drought+Warming+log(nosratio))^3,na.action=na.omit,day56only)

anova(m1a.nosz,m1b.nosz)

anova(m1a.nosz)

r.squaredGLMM(m1a.nosz)

m1a.nosz<-lme(N2O~(Plot+Drought+Warming+log(nosratio))^3,random=~1|fieldrep,na.action=na.omit,day56only)

anova(m1a.nosz)

#anova - acdS

m1a.acds<-lmer(N2O~(Plot+Drought+Warming+acdsratio)^3+(1|fieldrep),na.action=na.omit,day56only)

m1b.acds<-lm(N2O~(Plot+Drought+Warming+acdsratio)^3,na.action=na.omit,day56only)

anova(m1a.acds,m1b.acds)

anova(m1a.acds)

predacdsn2o<-predict(m1a.acds)

#

m1a.acds<-lmer(N2O~(Plot+Drought+Warming+acdsratio)^3+(1|fieldrep),na.action=na.omit,day56only)

## Decided not to use- mechanism unclear.

#anova - ITS

m1a.its<-lmer(N2O~(Plot+Drought+Warming+ITS)^3+(1|fieldrep),na.action=na.omit,day56only)

m1b.its<-lm(N2O~(Plot+Drought+Warming+ITS)^3,na.action=na.omit,day56only)

anova(m1a.its,m1b.its)

anova(m1b.its)

r.squaredGLMM(m1b.its)

#anova - X16s

m1a.16s<-lmer(N2O~(Plot+Drought+Warming+X16S)^3+(1|fieldrep),na.action=na.omit,day56only)

m1b.16s<-lm(N2O~(Plot+Drought+Warming+X16S)^3,na.action=na.omit,day56only)

anova(m1a.16s,m1b.16s)

anova(m1a.16s)

pred16sn2o<-predict(m1a.16s)

r.squaredGLMM(m1a.16s)

m1a.16s<-lme(N2O~(Plot+Drought+Warming+X16S)^3,random=~1|fieldrep,na.action=na.omit,day56only)

###############################################################

###############################################################

# CO2

#anova - nosZ

dotchart(day56only$nosratio)

m1a.nosz<-lmer(CO2~(Plot+Drought+Warming+log(nosratio))^3+(1|fieldrep),na.action=na.omit,day56only)

m1b.nosz<-lm(CO2~(Plot+Drought+Warming+log(nosratio))^3,na.action=na.omit,day56only)

anova(m1a.nosz,m1b.nosz)

anova(m1b.nosz)

r.squaredGLMM(m1b.nosz)

## Mechanism unclear

#anova - acdS

m1a.acds<-lmer(CO2~(Plot+Drought+Warming+acdsratio)^3+(1|fieldrep),na.action=na.omit,day56only)

m1b.acds<-lm(CO2~(Plot+Drought+Warming+acdsratio)^3,na.action=na.omit,day56only)

anova(m1a.acds,m1b.acds)

anova(m1b.acds)

r.squaredGLMM(m1b.acds)

#anova - ITS

m1a.its<-lmer(CO2~(Plot+Drought+Warming+ITS)^3+(1|fieldrep),na.action=na.omit,day56only)

m1b.its<-lm(CO2~(Plot+Drought+Warming+ITS)^3,na.action=na.omit,day56only)

anova(m1a.its,m1b.its)

anova(m1b.its)

r.squaredGLMM(m1b.its)

#anova - X16s

m1a.16s<-lmer(CO2~(Plot+Drought+Warming+X16S)^3+(1|fieldrep),na.action=na.omit,day56only)

m1b.16s<-lm(CO2~(Plot+Drought+Warming+X16S)^3,na.action=na.omit,day56only)

anova(m1a.16s,m1b.16s)

anova(m1b.16s)

pred16sco2<-predict(m1b.16s)

r.squaredGLMM(m1b.16s)

#anova - pmoA

m1a.pmoa<-lmer(CO2~(Plot+Drought+Warming+pmoaratio)^3+(1|fieldrep),na.action=na.omit,day56only)

m1b.pmoa<-lm(CO2~(Plot+Drought+Warming+pmoaratio)^3,na.action=na.omit,day56only)

anova(m1a.pmoa,m1b.pmoa)

anova(m1b.pmoa)

m1b.pmoa<-lm(CO2~(Plot+Drought+Warming+pmoaratio)^3,na.action=na.omit,day56only)

r.squaredGLMM(m1b.pmoa)

predpmoaco2<-predict(m1b.pmoa)

########################################################################################################

###############################################################

###############################################################

# Methane

#anova - acdS

m1a.acds<-lmer(CH4~(Plot+Drought+Warming+acdsratio)^3+(1|fieldrep),na.action=na.omit,day56only)

m1b.acds<-lm(CH4~(Plot+Drought+Warming+acdsratio)^3,na.action=na.omit,day56only)

anova(m1a.acds,m1b.acds)

anova(m1b.acds)

predacdsch4<-predict(m1b.acds)

r.squaredGLMM(m1b.acds)

#anova - ITS

m1a.its<-lmer(CH4~(Plot+Drought+Warming+ITS)^3+(1|fieldrep),na.action=na.omit,day56only)

m1b.its<-lm(CH4~(Plot+Drought+Warming+ITS)^3,na.action=na.omit,day56only)

anova(m1a.its,m1b.its)

anova(m1b.its)

r.squaredGLMM(m1b.its)

#anova - X16s

m1a.16s<-lmer(CH4~(Plot+Drought+Warming+X16S)^3+(1|fieldrep),na.action=na.omit,day56only)

m1b.16s<-lm(CH4~(Plot+Drought+Warming+X16S)^3,na.action=na.omit,day56only)

anova(m1a.16s,m1b.16s)

anova(m1b.16s)

r.squaredGLMM(m1b.16s)

#anova - pmoA

m1a.pmoa<-lmer(CH4~(Plot+Drought+Warming+pmoaratio)^3+(1|fieldrep),na.action=na.omit,day56only)

m1b.pmoa<-lm(CH4~(Plot+Drought+Warming+pmoaratio)^3,na.action=na.omit,day56only)

anova(m1a.pmoa,m1b.pmoa)

anova(m1b.pmoa)

predpmoach4<-predict(m1b.pmoa)

r.squaredGLMM(m1b.pmoa)

#####################################################

###########################################################################

#

require(latticeExtra)

## CODE FOR VERY SMALL NUMBER NOTATION

fancy_scientific <- function(l) {

# turn in to character string in scientific notation

l <- format(l, scientific = TRUE)

# quote the part before the exponent to keep all the digits

l <- gsub("^(.*)e", "'\\1'e", l)

# turn the 'e+' into plotmath format

l <- gsub("e", "%*%10^", l)

# return this as an expression

parse(text=l)

}

#

# Export as metafile 1000 x 800 on big screen

# Renaming data in table for use in single parameters & comparing sites

day56only$Plot<- factor(day56only$Plot,levels = c("2Cassiope", "3Eriophorum","1Ranunculus","4Saxifraga"))

levels(day56only$Plot) <- list('Latnjajaure Dry heath'="2Cassiope", 'Latnjajaure Tussock tundra'="3Eriophorum",'Latnjajaure Wet meadow'="1Ranunculus",'Val Bercla Alpine'="4Saxifraga")

day56only$Plot

day56only$Drought<- factor(day56only$Drought,levels = c("Wet", "Dry"))

levels(day56only$Drought) <- list('Control'="Wet", 'Drought'="Dry")

day56only$Drought

day56only$Warming<- factor(day56only$Warming,levels = c("C", "W"))

levels(day56only$Warming) <- list('Ambient'="C", 'Warmed'="W")

day56only$Warming

##############################################################################

# CO2 16S

day56only1<-cbind(day56only,pred16sco2)

#Latnjajaure dry heath

swed.dh<-day56only1[c(21:40),]

swed.dh_exp_CO2<-ggplot(swed.dh) +

theme_bw(base_size=16) +

scale_x_continuous(trans='log10', labels=fancy_scientific)+

aes(x = X16S, y =pred16sco2, colour=Drought,shape=Warming) +

geom_point(size=3) +

geom_smooth(method = "lm", alpha = .15, se=FALSE, fullrange=F, aes(colour=Drought, linetype=Warming))+

geom_hline(yintercept=0, color="darkgrey", linetype="dashed")+

theme(plot.title = element_blank(),

panel.grid.minor=element_blank(),

axis.text.x = element_text(angle = 45, hjust = 1),

panel.grid.major.y=element_blank(),

legend.position="none",

legend.title = element_blank(),

strip.background = element_blank())+

scale_color_manual(values = c("Control" = "darkblue", "Drought"="orange")) +

scale_shape_manual(values = c(16,1))+

ylim(-200,1300)+

ylab(expression(paste("Carbon dioxide flux mg " ~ m^2 ~ hr^-1))) +

theme_cowplot()+

xlab("16S bacterial gene abundance")+

facet_grid(~Plot)

swed.dh_exp_CO2

swed.dh_p2_CO2 <- ggplot(swed.dh, aes(x= X16S, group=Treatment, colour=Drought,linetype=Warming)) +

scale_x_continuous(trans='log10', labels=fancy_scientific)+

geom_density(adjust=1.5, alpha=.4,size=2)+

theme(plot.title = element_blank(),

panel.grid.minor=element_blank(),

panel.grid.major=element_blank(),

axis.text.y = element_text(angle = 45, hjust = 1),

panel.grid.major.y=element_blank(),

#legend.position="none",

legend.title = element_blank(),

strip.background = element_blank())+

facet_wrap(~Plot)+

scale_color_manual(values = c("Control" = "darkblue", "Drought"="orange")) +

theme_cowplot()+

theme(legend.position = 'none')+

xlab(element_blank())

swed.dh_p2_CO2

swed.dh_p1_CO2 <- ggplot(swed.dh, aes(x= pred16sco2, group=Treatment, colour=Drought,linetype=Warming)) +

#scale_y_continuous( labels=fancy_scientific)+

geom_density(adjust=1.5, alpha=.4,size=2)+

facet_grid(~Plot)+

theme(plot.title = element_blank(),

panel.grid.minor=element_blank(),

panel.grid.major=element_blank(),

axis.text.y = element_text(angle = 45, hjust = 1),

panel.grid.major.y=element_blank(),

#legend.position="none",

legend.title = element_blank(),

strip.background = element_blank())+

theme(legend.position = 'none')+

scale_color_manual(values = c("Control" = "darkblue", "Drought"="orange")) +

scale_shape_manual(values = c(16,1))+

xlim(-200,1300)+

theme_cowplot()+

xlab(element_blank())+

ylab(element_blank())+

geom_vline(xintercept=0, color="darkgrey", linetype="dashed")+

coord_flip()

swed.dh_p1_CO2

layout <- "

AAAA##

BBBBCC

BBBBCC

"

swed.dh_p2_CO2 + swed.dh_exp_CO2 + swed.dh_p1_CO2+plot_layout(design = layout,guides = 'collect')

## Export at 1000 x 750

#Latnjajaure tussock tundra

swed.tt<-day56only1[c(41:49,51:60),]

swed.tt_exp_CO2<-ggplot(swed.tt) +

theme_bw(base_size=16) +

scale_x_continuous(trans='log10', labels=fancy_scientific)+

aes(x = X16S, y = pred16sco2, colour=Drought,shape=Warming) +

geom_point(size=3) +

geom_smooth(method = "lm", alpha = .15, se=FALSE, fullrange=F, aes(colour=Drought, linetype=Warming))+

geom_hline(yintercept=0, color="darkgrey", linetype="dashed")+

theme(plot.title = element_blank(),

panel.grid.minor=element_blank(),

axis.text.x = element_text(angle = 45, hjust = 1),

panel.grid.major.y=element_blank(),

legend.position="none",

legend.title = element_blank(),

strip.background = element_blank())+

scale_color_manual(values = c("Control" = "darkblue", "Drought"="orange")) +

scale_shape_manual(values = c(16,1))+

ylab(expression(paste("Carbon dioxide flux mg " ~ m^2 ~ hr^-1))) +

ylim(-200,1300)+

theme_cowplot()+

xlab("16S bacterial gene abundance")+

facet_grid(~Plot)

swed.tt_exp_CO2

swed.tt_p2_CO2 <- ggplot(swed.tt, aes(x= X16S, group=Treatment, colour=Drought,linetype=Warming)) +

scale_x_continuous(trans='log10', labels=fancy_scientific)+

geom_density(adjust=1.5, alpha=.4,size=2)+

theme(plot.title = element_blank(),

panel.grid.minor=element_blank(),

panel.grid.major=element_blank(),

axis.text.y = element_text(angle = 45, hjust = 1),

panel.grid.major.y=element_blank(),

#legend.position="none",

legend.title = element_blank(),

strip.background = element_blank())+

facet_wrap(~Plot)+

scale_color_manual(values = c("Control" = "darkblue", "Drought"="orange")) +

theme_cowplot()+

theme(legend.position = 'none')+

xlab(element_blank())

swed.tt_p2_CO2

swed.tt_p1_CO2 <- ggplot(swed.tt, aes(x= pred16sco2, group=Treatment, colour=Drought,linetype=Warming)) +

#scale_y_continuous( labels=fancy_scientific)+

geom_density(adjust=1.5, alpha=.4,size=2)+

facet_grid(~Plot)+

theme(plot.title = element_blank(),

panel.grid.minor=element_blank(),

panel.grid.major=element_blank(),

axis.text.y = element_text(angle = 45, hjust = 1),

panel.grid.major.y=element_blank(),

#legend.position="none",

legend.title = element_blank(),

strip.background = element_blank())+

theme(legend.position = 'none')+

xlim(-200,1300)+

scale_color_manual(values = c("Control" = "darkblue", "Drought"="orange")) +

theme_cowplot()+

xlab(element_blank())+

ylab(element_blank())+

geom_vline(xintercept=0, color="darkgrey", linetype="dashed")+

coord_flip()

swed.tt_p1_CO2

layout <- "

AAAA##

BBBBCC

BBBBCC

"

swed.tt_p2_CO2 + swed.tt_exp_CO2 + swed.tt_p1_CO2+plot_layout(design = layout,guides = 'collect')

## Export at 1000 x 750

#Latnjajaure wet meadow

swed.wm<-day56only1[c(1:20),]

swed.wm_exp_CO2<-ggplot(swed.wm) +

theme_bw(base_size=16) +

scale_x_continuous(trans='log10', labels=fancy_scientific)+

aes(x = X16S, y = pred16sco2, colour=Drought, shape=Warming) +

geom_point(size=3) +

geom_smooth(method = "lm", alpha = .15, se=FALSE, fullrange=F, aes(colour=Drought, linetype=Warming))+

geom_hline(yintercept=0, color="darkgrey", linetype="dashed")+

theme(plot.title = element_blank(),

panel.grid.minor=element_blank(),

axis.text.x = element_text(angle = 45, hjust = 1),

panel.grid.major.y=element_blank(),

legend.position="none",

legend.title = element_blank(),

strip.background = element_blank())+

scale_color_manual(values = c("Control" = "darkblue", "Drought"="orange")) +

scale_shape_manual(values = c(16,1))+

ylab(expression(paste("Carbon dioxide flux mg " ~ m^2 ~ hr^-1))) +

ylim(-200,1300)+

theme_cowplot()+

xlab("16S bacterial gene abundance")+

facet_grid(~Plot)

swed.wm_exp_CO2

swed.wm_p2_CO2 <- ggplot(swed.wm, aes(x= X16S, group=Treatment, colour=Drought,linetype=Warming)) +

scale_x_continuous(trans='log10', labels=fancy_scientific)+

geom_density(adjust=1.5, alpha=.4,size=2)+

theme(plot.title = element_blank(),

panel.grid.minor=element_blank(),

panel.grid.major=element_blank(),

axis.text.y = element_text(angle = 45, hjust = 1),

panel.grid.major.y=element_blank(),

#legend.position="none",

legend.title = element_blank(),

strip.background = element_blank())+

facet_wrap(~Plot)+

scale_color_manual(values = c("Control" = "darkblue", "Drought"="orange")) +

theme_cowplot()+

theme(legend.position = 'none')+

xlab(element_blank())

swed.wm_p2_CO2

swed.wm_p1_CO2 <- ggplot(swed.wm, aes(x=pred16sco2, group=Treatment, colour=Drought,linetype=Warming)) +

#scale_y_continuous( labels=fancy_scientific)+

geom_density(adjust=1.5, alpha=.4,size=2)+

facet_grid(~Plot)+

theme(plot.title = element_blank(),

panel.grid.minor=element_blank(),

panel.grid.major=element_blank(),

axis.text.y = element_text(angle = 45, hjust = 1),

panel.grid.major.y=element_blank(),

#legend.position="none",

legend.title = element_blank(),

strip.background = element_blank())+

theme(legend.position = 'none')+

scale_color_manual(values = c("Control" = "darkblue", "Drought"="orange")) +

theme_cowplot()+

xlab(element_blank())+

xlim(-200,1300)+

ylab(element_blank())+

geom_vline(xintercept=0, color="darkgrey", linetype="dashed")+

coord_flip()

swed.wm_p1_CO2

layout <- "

AAAA##

BBBBCC

BBBBCC

"

swed.wm_p2_CO2 + swed.wm_exp_CO2 + swed.wm_p1_CO2+plot_layout(design = layout,guides = 'collect')

#Val Bercla alpine

swtz.a<-day56only1[c(61:96),]

swtz.a_exp_CO2<-ggplot(swtz.a) +

theme_bw(base_size=16) +

scale_x_continuous(trans='log10', labels=fancy_scientific)+

aes(x = X16S, y = pred16sco2, colour=Drought,shape=Warming) +

geom_point(size=3) +

geom_smooth(method = "lm", alpha = .15, se=FALSE, fullrange=F, aes(colour=Drought, linetype=Warming))+

geom_hline(yintercept=0, color="darkgrey", linetype="dashed")+

theme(plot.title = element_blank(),

panel.grid.minor=element_blank(),

axis.text.x = element_text(angle = 45, hjust = 1),

panel.grid.major.y=element_blank(),

legend.position="none",

legend.title = element_blank(),

strip.background = element_blank())+

scale_color_manual(values = c("Control" = "darkblue", "Drought"="orange")) +

scale_shape_manual(values = c(16, 1)) +

ylab(expression(paste("Carbon dioxide flux mg " ~ m^2 ~ hr^-1))) +

theme_cowplot()+

ylim(-200,1300)+

xlab("16S bacterial gene abundance")+

facet_grid(~Plot)

swtz.a_exp_CO2

swtz.a_p2_CO2 <- ggplot(swtz.a, aes(x= X16S, group=Treatment, colour=Drought,linetype=Warming)) +

scale_x_continuous(trans='log10', labels=fancy_scientific)+

geom_density(adjust=1.5, alpha=.4,size=2)+

theme(plot.title = element_blank(),

panel.grid.minor=element_blank(),

panel.grid.major=element_blank(),

axis.text.y = element_text(angle = 45, hjust = 1),

panel.grid.major.y=element_blank(),

#legend.position="none",

legend.title = element_blank(),

strip.background = element_blank())+

facet_wrap(~Plot)+

scale_color_manual(values = c("Control" = "darkblue", "Drought"="orange")) +

theme_cowplot()+

theme(legend.position = 'none')+

xlab(element_blank())

swtz.a_p2_CO2

swtz.a_p1_CO2 <- ggplot(swtz.a, aes(x= pred16sco2, group=Treatment, colour=Drought,linetype=Warming)) +

#scale_y_continuous( labels=fancy_scientific)+

geom_density(adjust=1.5, alpha=.4,size=2)+

facet_grid(~Plot)+

theme(plot.title = element_blank(),

panel.grid.minor=element_blank(),

panel.grid.major=element_blank(),

axis.text.y = element_text(angle = 45, hjust = 1),

panel.grid.major.y=element_blank(),

#legend.position="none",

legend.title = element_blank(),

strip.background = element_blank())+

theme(legend.position = 'none')+

xlim(-200,1300)+

scale_color_manual(values = c("Control" = "darkblue", "Drought"="orange")) +

theme_cowplot()+

xlab(element_blank())+

ylab(element_blank())+

geom_vline(xintercept=0, color="darkgrey", linetype="dashed")+

coord_flip()

swtz.a_p1_CO2

layout <- "

AAAA##

BBBBCC

BBBBCC

"

swtz.a_p2_CO2 + swtz.a_exp_CO2 + swtz.a_p1_CO2+plot_layout(design = layout,guides = 'collect')

##############################################################################

##################################################################################################

###################################################################################################

# CO2 pmoA

day56only1<-cbind(day56only,predpmoaco2)

#Dry heath

swed.dh<-day56only1[c(21:40),]

swed.dh_exp_CO2<-ggplot(swed.dh) +

theme_bw(base_size=16) +

# scale_x_continuous(labels=fancy_scientific)+

aes(x = pmoaratio, y = predpmoaco2, colour=Drought,linetype=Warming) +

geom_point(size=3) +

geom_smooth(method = "lm", alpha = .15, se=FALSE, fullrange=F, aes(colour=Drought,linetype=Warming))+

geom_hline(yintercept=0, color="darkgrey", linetype="dashed")+

theme(plot.title = element_blank(),

panel.grid.minor=element_blank(),

axis.text.x = element_text(angle = 45, hjust = 1),

panel.grid.major.y=element_blank(),

legend.position="none",

legend.title = element_blank(),

strip.background = element_blank())+

scale_color_manual(values = c("Control" = "darkblue", "Drought"="orange")) +

ylab(expression(paste("Carbon dioxide flux mg " ~ m^2 ~ hr^-1))) +

ylim(0,1000)+

xlim(0,0.005)+

theme_cowplot()+

xlab("pmoA: bacterial gene abundance")

swed.dh_exp_CO2

swed.dh_p2_CO2 <- ggplot(swed.dh, aes(x= pmoaratio, colour=Drought,linetype=Warming)) +

scale_x_continuous(labels=fancy_scientific)+

geom_density(adjust=1.5, alpha=.4,size=2)+

xlim(0,0.005)+

theme(plot.title = element_blank(),

panel.grid.minor=element_blank(),

panel.grid.major=element_blank(),

axis.text.y = element_text(angle = 45, hjust = 1),

panel.grid.major.y=element_blank(),

#legend.position="none",

legend.title = element_blank(),

strip.background = element_blank())+

scale_color_manual(values = c("Control" = "darkblue", "Drought"="orange")) +

theme_cowplot()+

theme(legend.position = 'none')+

xlab(element_blank())

swed.dh_p2_CO2

swed.dh_p1_CO2 <- ggplot(swed.dh, aes(x= predpmoaco2, colour=Drought,linetype=Warming)) +

#scale_y_continuous( labels=fancy_scientific)+

geom_density(adjust=1.5, alpha=.4,size=2)+

facet_grid(~Plot)+

theme(plot.title = element_blank(),

panel.grid.minor=element_blank(),

panel.grid.major=element_blank(),

axis.text.y = element_text(angle = 45, hjust = 1),

panel.grid.major.y=element_blank(),

#legend.position="none",

legend.title = element_blank(),

strip.background = element_blank())+

theme(legend.position = 'none')+

xlim(0,1000)+

scale_color_manual(values = c("Control" = "darkblue","Drought"="orange")) +

theme_cowplot()+

xlab(element_blank())+

ylab(element_blank())+

geom_vline(xintercept=0, color="darkgrey", linetype="dashed")+

coord_flip()

swed.dh_p1_CO2

layout <- "

AAAA##

BBBBCC

BBBBCC

"

swed.dh_p2_CO2 + swed.dh_exp_CO2 + swed.dh_p1_CO2+plot_layout(design = layout,guides = 'collect')

## Export at 1000 x 750

#Latnjajaure tussock tundra

swed.tt<-day56only1[c(41:60),]

swed.tt_exp_CO2<-ggplot(swed.tt) +

theme_bw(base_size=16) +

scale_x_continuous( labels=fancy_scientific)+

aes(x = pmoaratio, y = predpmoaco2, colour=Drought,linetype=Warming) +

geom_point(size=3) +

geom_smooth(method = "lm", alpha = .15, se=FALSE, fullrange=F, aes(colour=Drought,linetype=Warming))+

geom_hline(yintercept=0, color="darkgrey", linetype="dashed")+

theme(plot.title = element_blank(),

panel.grid.minor=element_blank(),

axis.text.x = element_text(angle = 45, hjust = 1),

panel.grid.major.y=element_blank(),

legend.position="none",

legend.title = element_blank(),

strip.background = element_blank())+

scale_color_manual(values = c("Control" = "darkblue", "Drought"="orange")) +

ylab(expression(paste("Carbon dioxide flux mg " ~ m^2 ~ hr^-1))) +

xlim(0,0.005)+

ylim(0,1000)+

theme_cowplot()+

xlab("pmoA: bacterial gene abundance")

swed.tt_exp_CO2

swed.tt_p2_CO2 <- ggplot(swed.tt, aes(x= pmoaratio, colour=Drought,linetype=Warming)) +

scale_x_continuous(labels=fancy_scientific)+

geom_density(adjust=1.5, alpha=.4,size=2)+

theme(plot.title = element_blank(),

panel.grid.minor=element_blank(),

panel.grid.major=element_blank(),

axis.text.y = element_text(angle = 45, hjust = 1),

panel.grid.major.y=element_blank(),

#legend.position="none",

legend.title = element_blank(),

strip.background = element_blank())+

scale_color_manual(values = c("Control" = "darkblue", "Drought"="orange")) +

xlim(0,0.005)+

theme_cowplot()+

theme(legend.position = 'none')+

xlab(element_blank())

swed.tt_p2_CO2

swed.tt_p1_CO2 <- ggplot(swed.tt, aes(x= predpmoaco2, colour=Drought,linetype=Warming)) +

#scale_y_continuous( labels=fancy_scientific)+

geom_density(adjust=1.5, alpha=.4,size=2)+

facet_grid(~Plot)+

theme(plot.title = element_blank(),

panel.grid.minor=element_blank(),

panel.grid.major=element_blank(),

axis.text.y = element_text(angle = 45, hjust = 1),

panel.grid.major.y=element_blank(),

#legend.position="none",

legend.title = element_blank(),

strip.background = element_blank())+

theme(legend.position = 'none')+

scale_color_manual(values = c("Control" = "darkblue", "Drought"="orange")) +

theme_cowplot()+

xlim(0,1000)+

xlab(element_blank())+

ylab(element_blank())+

geom_vline(xintercept=0, color="darkgrey", linetype="dashed")+

coord_flip()

swed.tt_p1_CO2

layout <- "

AAAA##

BBBBCC

BBBBCC

"

swed.tt_p2_CO2 + swed.tt_exp_CO2 + swed.tt_p1_CO2+plot_layout(design = layout,guides = 'collect')

## Export at 1000 x 750

#Latnjajaure wet meadow

swed.wm<-day56only1[c(1:20),]

swed.wm_exp_CO2<-ggplot(swed.wm) +

theme_bw(base_size=16) +

scale_x_continuous( labels=fancy_scientific)+

aes(x = pmoaratio, y = predpmoaco2, colour=Drought,linetype=Warming) +

geom_point(size=3) +

geom_smooth(method = "lm", alpha = .15, se=FALSE, fullrange=F, aes(colour=Drought,linetype=Warming))+

geom_hline(yintercept=0, color="darkgrey", linetype="dashed")+

theme(plot.title = element_blank(),

panel.grid.minor=element_blank(),

axis.text.x = element_text(angle = 45, hjust = 1),

panel.grid.major.y=element_blank(),

legend.position="none",

legend.title = element_blank(),

strip.background = element_blank())+

scale_color_manual(values = c("Control" = "darkblue", "Drought"="orange")) +

ylab(expression(paste("Carbon dioxide flux mg " ~ m^2 ~ hr^-1))) +

xlim(0,0.005)+

ylim(0,1000)+

theme_cowplot()+

xlab("pmoA: bacterial gene abundance")

swed.wm_exp_CO2

swed.wm_p2_CO2 <- ggplot(swed.wm, aes(x= pmoaratio, colour=Drought,linetype=Warming)) +

scale_x_continuous(labels=fancy_scientific)+

geom_density(adjust=1.5, alpha=.4,size=2)+

theme(plot.title = element_blank(),

panel.grid.minor=element_blank(),

panel.grid.major=element_blank(),

axis.text.y = element_text(angle = 45, hjust = 1),

panel.grid.major.y=element_blank(),

#legend.position="none",

legend.title = element_blank(),

strip.background = element_blank())+

scale_color_manual(values = c("Control" = "darkblue", "Drought"="orange")) +

xlim(0,0.005)+

theme_cowplot()+

theme(legend.position = 'none')+

xlab(element_blank())

swed.wm_p2_CO2

swed.wm_p1_CO2 <- ggplot(swed.wm, aes(x= predpmoaco2, colour=Drought,linetype=Warming)) +

#scale_y_continuous( labels=fancy_scientific)+

geom_density(adjust=1.5, alpha=.4,size=2)+

facet_grid(~Plot)+

theme(plot.title = element_blank(),

panel.grid.minor=element_blank(),

panel.grid.major=element_blank(),

axis.text.y = element_text(angle = 45, hjust = 1),

panel.grid.major.y=element_blank(),

#legend.position="none",

legend.title = element_blank(),

strip.background = element_blank())+

theme(legend.position = 'none')+

scale_color_manual(values = c("Control" = "darkblue", "Drought"="orange")) +

theme_cowplot()+

ylim(0,0.01)+

xlab(element_blank())+

ylab(element_blank())+

geom_vline(xintercept=0, color="darkgrey", linetype="dashed")+

coord_flip()

swed.wm_p1_CO2

layout <- "

AAAA##

BBBBCC

BBBBCC

"

swed.wm_p2_CO2 + swed.wm_exp_CO2 + swed.wm_p1_CO2+plot_layout(design = layout,guides = 'collect')

#Val Bercla alpine

swtz.a<-day56only1[c(61:96),]

swtz.a_exp_CO2<-ggplot(swtz.a) +

theme_bw(base_size=16) +

scale_x_continuous( labels=fancy_scientific)+

aes(x = pmoaratio, y = predpmoaco2, colour=Drought,linetype=Warming) +

geom_point(size=3) +

geom_smooth(method = "lm", alpha = .15, se=FALSE, fullrange=F, aes(colour=Drought,linetype=Warming))+

geom_hline(yintercept=0, color="darkgrey", linetype="dashed")+

theme(plot.title = element_blank(),

panel.grid.minor=element_blank(),

axis.text.x = element_text(angle = 45, hjust = 1),

panel.grid.major.y=element_blank(),

legend.position="none",

legend.title = element_blank(),

strip.background = element_blank())+

scale_color_manual(values = c("Control" = "darkblue", "Drought"="orange")) +

ylab(expression(paste("Carbon dioxide flux mg " ~ m^2 ~ hr^-1))) +

xlim(0,0.005)+

ylim(0,1000)+

theme_cowplot()+

xlab("pmoA: bacterial gene abundance")

swtz.a_exp_CO2

swtz.a_p2_CO2 <- ggplot(swtz.a, aes(x= pmoaratio, colour=Drought,linetype=Warming)) +

scale_x_continuous(labels=fancy_scientific)+

geom_density(adjust=1.5, alpha=.4,size=2)+

theme(plot.title = element_blank(),

panel.grid.minor=element_blank(),

panel.grid.major=element_blank(),

axis.text.y = element_text(angle = 45, hjust = 1),

panel.grid.major.y=element_blank(),

#legend.position="none",

legend.title = element_blank(),

strip.background = element_blank())+

xlim(0,0.005)+

scale_color_manual(values = c("Control" = "darkblue", "Drought"="orange")) +

theme_cowplot()+

theme(legend.position = 'none')+

xlab(element_blank())

swtz.a_p2_CO2

swtz.a_p1_CO2 <- ggplot(swtz.a, aes(x= predpmoaco2, colour=Drought,linetype=Warming)) +

#scale_y_continuous( labels=fancy_scientific)+

geom_density(adjust=1.5, alpha=.4,size=2)+

facet_grid(~Plot)+

theme(plot.title = element_blank(),

panel.grid.minor=element_blank(),

panel.grid.major=element_blank(),

axis.text.y = element_text(angle = 45, hjust = 1),

panel.grid.major.y=element_blank(),

#legend.position="none",

legend.title = element_blank(),

strip.background = element_blank())+

theme(legend.position = 'none')+

scale_color_manual(values = c("Control" = "darkblue", "Drought"="orange")) +

theme_cowplot()+

xlim(0,950)+

xlab(element_blank())+

ylab(element_blank())+

geom_vline(xintercept=0, color="darkgrey", linetype="dashed")+

coord_flip()

swtz.a_p1_CO2

layout <- "

AAAA##

BBBBCC

BBBBCC

"

swtz.a_p2_CO2 + swtz.a_exp_CO2 + swtz.a_p1_CO2+plot_layout(design = layout,guides = 'collect')

##############################################################################

##################################################################################################

###################################################################################################

##############################################################################

# N2O 16S

day56only1<-cbind(day56only,pred16sn2o)

#Wet meadow

swed.wm<-day56only1[c(1:20),]

swed.wm_exp_n2o<-ggplot(swed.wm) +

theme_bw(base_size=16) +

scale_x_continuous(trans='log10', labels=fancy_scientific)+

aes(x = X16S, y =pred16sn2o,colour=Drought, shape=Warming) +

geom_point(size=3) +

geom_smooth(method = "lm", alpha = .15, se=FALSE, fullrange=F, aes(colour=Drought,linetype=Warming))+

geom_hline(yintercept=0, color="darkgrey", linetype="dashed")+

theme(plot.title = element_blank(),

panel.grid.minor=element_blank(),

axis.text.x = element_text(angle = 45, hjust = 1),

panel.grid.major.y=element_blank(),

legend.position="none",

legend.title = element_blank(),

strip.background = element_blank())+

scale_color_manual(values = c("Control" = "darkblue", "Drought"="orange")) +

ylim(-50,150)+

xlim(1000000,550000000)+

ylab(expression(paste("Nitrous oxide flux ug " ~ m^2 ~ hr^-1))) +

theme_cowplot()+

xlab("16S bacterial gene abundance")+

facet_grid(~Plot)

swed.wm_exp_n2o

swed.wm_p2_N2O <- ggplot(swed.wm, aes(x= X16S, colour=Drought, linetype=Warming)) +

geom_density(adjust=1.5, alpha=.4,size=2)+

facet_grid(~Plot)+

theme(plot.title = element_blank(),

panel.grid.minor=element_blank(),

panel.grid.major=element_blank(),

axis.text.y = element_text(angle = 45, hjust = 1),

panel.grid.major.y=element_blank(),

#legend.position="none",

legend.title = element_blank(),

strip.background = element_blank())+

theme(legend.position = 'none')+

scale_color_manual(values = c("Control" = "darkblue", "Drought"="orange")) +

theme_cowplot()+

xlim(1000000,550000000)+

xlab(element_blank())+

ylab(element_blank())+

geom_vline(xintercept=0, color="darkgrey", linetype="dashed")#

swed.wm_p2_N2O

swed.wm_p1_N2O <- ggplot(swed.wm, aes(x= pred16sn2o, colour=Drought, linetype=Warming)) +

#scale_y_continuous( labels=fancy_scientific)+

geom_density(adjust=1.5, alpha=.4,size=2)+

theme(plot.title = element_blank(),

panel.grid.minor=element_blank(),

panel.grid.major=element_blank(),

axis.text.y = element_text(angle = 45, hjust = 1),

panel.grid.major.y=element_blank(),

#legend.position="none",

legend.title = element_blank(),

strip.background = element_blank())+

theme(legend.position = 'none')+

scale_color_manual(values = c("Control" = "darkblue", "Drought"="orange")) +

theme_cowplot()+

xlim(-50,150)+

xlab(element_blank())+

ylab(element_blank())+

geom_vline(xintercept=0, color="darkgrey", linetype="dashed")+

coord_flip()

swed.wm_p1_N2O

layout <- "

AAAA##

BBBBCC

BBBBCC

"

swed.wm_p2_N2O + swed.wm_exp_n2o + swed.wm_p1_N2O+plot_layout(design = layout,guides = 'collect')

## Export at 1000 x 750

#Latnjajaure dry heath

swed.dh<-day56only1[c(21:40),]

swed.dh_exp_n2o<-ggplot(swed.dh) +theme_bw(base_size=16) +

scale_x_continuous(trans='log10', labels=fancy_scientific)+

aes(x = X16S, y =pred16sn2o,colour=Drought, shape=Warming) +

geom_point(size=3) +

geom_smooth(method = "lm", alpha = .15, se=FALSE, fullrange=F, aes(colour=Drought,linetype=Warming))+

geom_hline(yintercept=0, color="darkgrey", linetype="dashed")+

theme(plot.title = element_blank(),

panel.grid.minor=element_blank(),

axis.text.x = element_text(angle = 45, hjust = 1),

panel.grid.major.y=element_blank(),

legend.position="none",

legend.title = element_blank(),

strip.background = element_blank())+

scale_color_manual(values = c("Control" = "darkblue", "Drought"="orange")) +

ylim(-50,150)+

xlim(1000000,550000000)+

ylab(expression(paste("Nitrous oxide flux ug " ~ m^2 ~ hr^-1))) +

theme_cowplot()+

xlab("16S bacterial gene abundance")+

facet_grid(~Plot)

swed.dh_exp_n2o

swed.dh_p2_N2O <- ggplot(swed.dh, aes(x= X16S,colour=Drought, linetype=Warming)) +

scale_x_continuous(labels=fancy_scientific)+

geom_density(adjust=1.5, alpha=.4,size=2)+

theme(plot.title = element_blank(),

panel.grid.minor=element_blank(),

panel.grid.major=element_blank(),

axis.text.y = element_text(angle = 45, hjust = 1),

panel.grid.major.y=element_blank(),

#legend.position="none",

legend.title = element_blank(),

strip.background = element_blank())+

facet_wrap(~Plot)+

scale_shape_manual(values = c(16,1))+

scale_color_manual(values = c("Control" = "darkblue", "Drought"="orange")) +

xlim(1000000,550000000)+

theme_cowplot()+

theme(legend.position = 'none')+

xlab(element_blank())

swed.dh_p2_N2O

swed.dh_p1_N2O <- ggplot(swed.dh, aes(x= pred16sn2o,colour=Drought, linetype=Warming)) +

#scale_y_continuous( labels=fancy_scientific)+

geom_density(adjust=1.5, alpha=.4,size=2)+

facet_grid(~Plot)+

theme(plot.title = element_blank(),

panel.grid.minor=element_blank(),

panel.grid.major=element_blank(),

axis.text.y = element_text(angle = 45, hjust = 1),

panel.grid.major.y=element_blank(),

#legend.position="none",

legend.title = element_blank(),

strip.background = element_blank())+

theme(legend.position = 'none')+

scale_shape_manual(values = c(16,1))+ theme_cowplot()+

scale_color_manual(values = c("Control" = "darkblue", "Drought"="orange")) +

xlim(-50,150)+

xlab(element_blank())+

ylab(element_blank())+

geom_vline(xintercept=0, color="darkgrey", linetype="dashed")+

coord_flip()

swed.dh_p1_N2O

layout <- "

AAAA##

BBBBCC

BBBBCC

"

swed.dh_p2_N2O + swed.dh_exp_n2o + swed.dh_p1_N2O+plot_layout(design = layout,guides = 'collect')

## Export at 1000 x 750

#Latnjajaure tussock tundra

swed.tt<-day56only1[c(41:60),]

swed.tt_exp_N2O<-ggplot(swed.tt) +

theme_bw(base_size=16) +

scale_x_continuous(trans='log10', labels=fancy_scientific)+

aes(x = X16S, y =pred16sn2o, colour=Drought,shape=Warming) +

geom_point(size=3) +

geom_smooth(method = "lm", alpha = .15, se=FALSE, fullrange=F, aes(colour=Drought,linetype=Warming))+

geom_hline(yintercept=0, color="darkgrey", linetype="dashed")+

theme(plot.title = element_blank(),

panel.grid.minor=element_blank(),

axis.text.x = element_text(angle = 45, hjust = 1),

panel.grid.major.y=element_blank(),

legend.position="none",

legend.title = element_blank(),

strip.background = element_blank())+

scale_color_manual(values = c("Control" = "darkblue", "Drought"="orange")) +

scale_shape_manual(values = c(16,1))+

ylim(-50,150)+

xlim(1000000,550000000)+

ylab(expression(paste("Nitrous oxide flux ug " ~ m^2 ~ hr^-1))) +

theme_cowplot()+

xlab("16S bacterial gene abundance")

swed.tt_exp_N2O

swed.tt_p2_N2O <- ggplot(swed.tt, aes(x= X16S,colour=Drought, linetype=Warming)) +

scale_x_continuous(labels=fancy_scientific)+

geom_density(adjust=1.5, alpha=.4,size=2)+

theme(plot.title = element_blank(),

panel.grid.minor=element_blank(),

panel.grid.major=element_blank(),

axis.text.y = element_text(angle = 45, hjust = 1),

panel.grid.major.y=element_blank(),

#legend.position="none",

legend.title = element_blank(),

strip.background = element_blank())+

scale_color_manual(values = c("Control" = "darkblue", "Drought"="orange")) +

xlim(1000000,550000000)+

scale_shape_manual(values = c(16,1))+

theme_cowplot()+

theme(legend.position = 'none')+

xlab(element_blank())

swed.tt_p2_N2O

swed.tt_p1_N2O <- ggplot(swed.tt, aes(x= pred16sn2o,colour=Drought, linetype=Warming)) +

#scale_y_continuous( labels=fancy_scientific)+

geom_density(adjust=1.5, alpha=.4,size=2)+

facet_grid(~Plot)+

theme(plot.title = element_blank(),

panel.grid.minor=element_blank(),

panel.grid.major=element_blank(),

axis.text.y = element_text(angle = 45, hjust = 1),

panel.grid.major.y=element_blank(),

#legend.position="none",

legend.title = element_blank(),

strip.background = element_blank())+

theme(legend.position = 'none')+

scale_color_manual(values = c("Control" = "darkblue", "Drought"="orange")) +

scale_shape_manual(values = c(16,1))+

theme_cowplot()+

xlab(element_blank())+

ylab(element_blank())+

xlim(-50,150)+

geom_vline(xintercept=0, color="darkgrey", linetype="dashed")+

coord_flip()

swed.tt_p1_N2O

layout <- "

AAAA##

BBBBCC

BBBBCC

"

swed.tt_p2_N2O + swed.tt_exp_N2O + swed.tt_p1_N2O+plot_layout(design = layout,guides = 'collect')

#Val Bercla alpine

swtz.a<-day56only1[c(61:96),]

swtz.a_exp_N2O<-ggplot(swtz.a) +

theme_bw(base_size=16) +

scale_x_continuous(trans='log10', labels=fancy_scientific)+

aes(x = X16S, y =pred16sn2o, colour=Drought,shape=Warming) +

geom_point(size=3) +

geom_smooth(method = "lm", alpha = .15, se=FALSE, fullrange=F, aes(colour=Drought,linetype=Warming))+

geom_hline(yintercept=0, color="darkgrey", linetype="dashed")+

theme(plot.title = element_blank(),

panel.grid.minor=element_blank(),

axis.text.x = element_text(angle = 45, hjust = 1),

panel.grid.major.y=element_blank(),

legend.position="none",

legend.title = element_blank(),

strip.background = element_blank())+

scale_color_manual(values = c("Control" = "darkblue", "Drought"="orange")) +

scale_shape_manual(values = c(16,1))+

ylim(-50,150)+

xlim(1000000,550000000)+

ylab(expression(paste("Nitrous oxide flux ug " ~ m^2 ~ hr^-1))) +

theme_cowplot()+

xlab("16S bacterial gene abundance")

swtz.a_exp_N2O

swtz.a_p2_N2O <- ggplot(swtz.a, aes(x= X16S, colour=Drought,linetype=Warming)) +

scale_x_continuous(labels=fancy_scientific)+

geom_density(adjust=1.5, alpha=.4,size=2)+

theme(plot.title = element_blank(),

panel.grid.minor=element_blank(),

panel.grid.major=element_blank(),

axis.text.y = element_text(angle = 45, hjust = 1),

panel.grid.major.y=element_blank(),

#legend.position="none",

legend.title = element_blank(),

strip.background = element_blank())+

scale_color_manual(values = c("Control" = "darkblue", "Drought"="orange")) +

xlim(1000000,550000000)+

theme_cowplot()+

theme(legend.position = 'none')+

xlab(element_blank())

swtz.a_p2_N2O

swtz.a_p1_N2O <- ggplot(swtz.a, aes(x= pred16sn2o,colour=Drought, linetype=Warming)) +

#scale_y_continuous( labels=fancy_scientific)+

geom_density(adjust=1.5, alpha=.4,size=2)+

theme(plot.title = element_blank(),

panel.grid.minor=element_blank(),

panel.grid.major=element_blank(),

axis.text.y = element_text(angle = 45, hjust = 1),

panel.grid.major.y=element_blank(),

#legend.position="none",

legend.title = element_blank(),

strip.background = element_blank())+

theme(legend.position = 'none')+

scale_color_manual(values = c("Control" = "darkblue", "Drought"="orange")) +

theme_cowplot()+

xlim(-50,150)+

xlab(element_blank())+

ylab(element_blank())+

geom_vline(xintercept=0, color="darkgrey", linetype="dashed")+

coord_flip()

swtz.a_p1_N2O

layout <- "

AAAA##

BBBBCC

BBBBCC

"

swtz.a_p2_N2O + swtz.a_exp_N2O + swtz.a_p1_N2O+plot_layout(design = layout,guides = 'collect')

##################################################################################################

##

############

##################################################################################################

###################################################################################################

##############################################################################

# CH4 acdS- UNUSED

hist(CH4)

day56only1<-cbind(day56only,predacdsch4)

#Dry heath

swed.dh<-day56only1[c(21:40),]

swed.dh_exp_CH4<-ggplot(swed.dh) +

theme_bw(base_size=16) +

scale_x_continuous( labels=fancy_scientific)+

aes(x = pmoaratio, y = predacdsch4, colour=Drought,shape=Warming) +

geom_point(size=3) +

geom_smooth(method = "lm", alpha = .15, se=FALSE, fullrange=F, aes(colour=Drought, linetype=Warming))+

geom_hline(yintercept=0, color="darkgrey", linetype="dashed")+

theme(plot.title = element_blank(),

panel.grid.minor=element_blank(),

axis.text.x = element_text(angle = 45, hjust = 1),

panel.grid.major.y=element_blank(),

legend.position="none",

legend.title = element_blank(),

strip.background = element_blank())+

scale_color_manual(values = c("Control" = "darkgrey", "Drought"="black")) +

scale_shape_manual(values = c(16,1))+

ylab(expression(paste("Methane flux mg " ~ m^2 ~ hr^-1))) +

ylim(-450,650)+

xlim(0,0.001)+

theme_cowplot()+

xlab("acdS:16S ratio gene abundance")+

facet_grid(~Plot)

swed.dh_exp_CH4

swed.dh_p2_CH4 <- ggplot(swed.dh, aes(x= pmoaratio, group=Treatment, colour=Drought)) +

scale_x_continuous( labels=fancy_scientific)+

geom_density(adjust=1.5, alpha=.4,size=2)+

theme(plot.title = element_blank(),

panel.grid.minor=element_blank(),

panel.grid.major=element_blank(),

axis.text.y = element_text(angle = 45, hjust = 1),

panel.grid.major.y=element_blank(),

#legend.position="none",

legend.title = element_blank(),

strip.background = element_blank())+

facet_wrap(~Plot)+

xlim(0,0.001)+

scale_color_manual(values = c("Control" = "darkgrey", "Drought"="black")) +

theme_cowplot()+

theme(legend.position = 'none')+

xlab(element_blank())

swed.dh_p2_CH4

swed.dh_p1_CH4 <- ggplot(swed.dh, aes(x= predacdsch4, group=Treatment, colour=Drought)) +

#scale_y_continuous( labels=fancy_scientific)+

geom_density(adjust=1.5, alpha=.4,size=2)+

theme(plot.title = element_blank(),

panel.grid.minor=element_blank(),

panel.grid.major=element_blank(),

axis.text.y = element_text(angle = 45, hjust = 1),

panel.grid.major.y=element_blank(),

#legend.position="none",

legend.title = element_blank(),

strip.background = element_blank())+

theme(legend.position = 'none')+

scale_color_manual(values = c("Control" = "darkgrey", "Drought"="black")) +

theme_cowplot()+

xlim(-450,650)+

xlab(element_blank())+

ylab(element_blank())+

geom_vline(xintercept=0, color="darkgrey", linetype="dashed")+

coord_flip()

swed.dh_p1_CH4

layout <- "

AAAA##

BBBBCC

BBBBCC

"

swed.dh_p2_CH4 + swed.dh_exp_CH4 + swed.dh_p1_CH4+plot_layout(design = layout,guides = 'collect')

## Export at 1000 x 750

#Latnjajaure tussock tundra

swed.tt<-day56only1[c(41:60),]

swed.tt_exp_CH4<-ggplot(swed.tt) +

theme_bw(base_size=16) +

scale_x_continuous( labels=fancy_scientific)+

aes(x = acdsratio, y = predacdsch4, colour=Drought,shape=Warming) +

geom_point(size=3) +

geom_smooth(method = "lm", alpha = .15, se=FALSE, fullrange=F, aes(colour=Drought, linetype=Warming))+

geom_hline(yintercept=0, color="darkgrey", linetype="dashed")+

theme(plot.title = element_blank(),

panel.grid.minor=element_blank(),

axis.text.x = element_text(angle = 45, hjust = 1),

panel.grid.major.y=element_blank(),

legend.position="none",

legend.title = element_blank(),

strip.background = element_blank())+

scale_color_manual(values = c("Control" = "darkgrey", "Drought"="black")) +

scale_shape_manual(values = c(16,1))+

ylab(expression(paste("Methane flux mg " ~ m^2 ~ hr^-1))) +

theme_cowplot()+

ylim(-450,650)+

xlim(0,0.00001)+

xlab("acdS:16S ratio gene abundance")

swed.tt_exp_CH4

swed.tt_p2_CH4 <- ggplot(swed.tt, aes(x= acdsratio, group=Treatment, colour=Drought)) +

scale_x_continuous(labels=fancy_scientific)+

geom_density(adjust=1.5, alpha=.4,size=2)+

theme(plot.title = element_blank(),

panel.grid.minor=element_blank(),

panel.grid.major=element_blank(),

axis.text.y = element_text(angle = 45, hjust = 1),

panel.grid.major.y=element_blank(),

#legend.position="none",

legend.title = element_blank(),

strip.background = element_blank())+

scale_color_manual(values = c("Control" = "darkgrey", "Drought"="black")) +

xlim(0,0.00001)+

theme_cowplot()+

theme(legend.position = 'none')+

xlab(element_blank())

swed.tt_p2_CH4

swed.tt_p1_CH4 <- ggplot(swed.tt, aes(x= predacdsch4, group=Treatment, colour=Drought)) +

#scale_y_continuous( labels=fancy_scientific)+

geom_density(adjust=1.5, alpha=.4,size=2)+

theme(plot.title = element_blank(),

panel.grid.minor=element_blank(),

panel.grid.major=element_blank(),

axis.text.y = element_text(angle = 45, hjust = 1),

panel.grid.major.y=element_blank(),

#legend.position="none",

legend.title = element_blank(),

strip.background = element_blank())+

theme(legend.position = 'none')+

scale_color_manual(values = c("Control" = "darkgrey", "Drought"="black")) +

theme_cowplot()+

xlim(-450,650)+

xlab(element_blank())+

ylab(element_blank())+

geom_vline(xintercept=0, color="darkgrey", linetype="dashed")+

coord_flip()

swed.tt_p1_CH4

layout <- "

AAAA##

BBBBCC

BBBBCC

"

swed.tt_p2_CH4 + swed.tt_exp_CH4 + swed.tt_p1_CH4+plot_layout(design = layout,guides = 'collect')

## Export at 1000 x 750

#Latnjajaure wet meadow

swed.wm<-day56only1[c(1:20),]

swed.wm_exp_CH4<-ggplot(swed.wm) +

theme_bw(base_size=16) +

scale_x_continuous( labels=fancy_scientific)+

aes(x =acdsratio , y = predacdsch4, colour=Drought,shape=Warming) +

geom_point(size=3) +

geom_smooth(method = "lm", alpha = .15, se=FALSE, fullrange=F, aes(colour=Drought, linetype=Warming))+

geom_hline(yintercept=0, color="darkgrey", linetype="dashed")+

theme(plot.title = element_blank(),

panel.grid.minor=element_blank(),

axis.text.x = element_text(angle = 45, hjust = 1),

panel.grid.major.y=element_blank(),

legend.position="none",

legend.title = element_blank(),

strip.background = element_blank())+

ylim(-450,650)+

xlim(0,0.001)+

scale_color_manual(values = c("Control" = "darkgrey", "Drought"="black")) +

scale_shape_manual(values = c(16,1))+

ylab(expression(paste("Methane flux mg " ~ m^2 ~ hr^-1))) +

theme_cowplot()+

xlab("acdS:16S ratio gene abundance")+

facet_grid(~Plot)

swed.wm_exp_CH4

swed.wm_p2_CH4 <- ggplot(swed.wm, aes(x= acdsratio, group=Treatment, colour=Drought)) +

scale_x_continuous(labels=fancy_scientific)+

geom_density(adjust=1.5, alpha=.4,size=2)+

theme(plot.title = element_blank(),

panel.grid.minor=element_blank(),

panel.grid.major=element_blank(),

axis.text.y = element_text(angle = 45, hjust = 1),

panel.grid.major.y=element_blank(),

#legend.position="none",

legend.title = element_blank(),

strip.background = element_blank())+

xlim(0,.001)+

facet_wrap(~Plot)+

scale_color_manual(values = c("Control" = "darkgrey", "Drought"="black")) +

theme_cowplot()+

theme(legend.position = 'none')+

xlab(element_blank())

swed.wm_p2_CH4

swed.wm_p1_CH4 <- ggplot(swed.wm, aes(x= predacdsch4, group=Treatment, colour=Drought)) +

#scale_y_continuous( labels=fancy_scientific)+

geom_density(adjust=1.5, alpha=.4,size=2)+

facet_grid(~Plot)+

theme(plot.title = element_blank(),

panel.grid.minor=element_blank(),

panel.grid.major=element_blank(),

axis.text.y = element_text(angle = 45, hjust = 1),

panel.grid.major.y=element_blank(),

#legend.position="none",

legend.title = element_blank(),

strip.background = element_blank())+

theme(legend.position = 'none')+

scale_color_manual(values = c("Control" = "darkgrey", "Drought"="black")) +

theme_cowplot()+

xlab(element_blank())+

ylab(element_blank())+

xlim(-450,650)+

ylim(0,0.01)+

geom_vline(xintercept=0, color="darkgrey", linetype="dashed")+

coord_flip()

swed.wm_p1_CH4

layout <- "

AAAA##

BBBBCC

BBBBCC

"

swed.wm_p2_CH4 + swed.wm_exp_CH4 + swed.wm_p1_CH4+plot_layout(design = layout,guides = 'collect')

#Val Bercla alpine

swtz.a<-day56only1[c(61:96),]

swtz.a_exp_CH4<-ggplot(swtz.a) +

theme_bw(base_size=16) +

scale_x_continuous( labels=fancy_scientific)+

aes(x =acdsratio , y = predacdsch4, colour=Drought,shape=Warming) +

geom_point(size=3) +

geom_smooth(method = "lm", alpha = .15, se=FALSE, fullrange=F, aes(colour=Drought, linetype=Warming))+

geom_hline(yintercept=0, color="darkgrey", linetype="dashed")+

ylim(-450,650)+

xlim(0,0.0001)+

theme(plot.title = element_blank(),

panel.grid.minor=element_blank(),

axis.text.x = element_text(angle = 45, hjust = 1),

panel.grid.major.y=element_blank(),

legend.position="none",

legend.title = element_blank(),

strip.background = element_blank())+

scale_color_manual(values = c("Control" = "darkgrey", "Drought"="black")) +

scale_shape_manual(values = c(16,1))+

ylab(expression(paste("Methane flux mg " ~ m^2 ~ hr^-1))) +

theme_cowplot()+

xlab("acdS:16S ratio gene abundance")+

facet_grid(~Plot)

swtz.a_exp_CH4

swtz.a_p2_CH4 <- ggplot(swtz.a, aes(x= acdsratio, group=Treatment, colour=Drought)) +

scale_x_continuous(labels=fancy_scientific)+

geom_density(adjust=1.5, alpha=.4,size=2)+

theme(plot.title = element_blank(),

panel.grid.minor=element_blank(),

panel.grid.major=element_blank(),

axis.text.y = element_text(angle = 45, hjust = 1),

panel.grid.major.y=element_blank(),

#legend.position="none",

legend.title = element_blank(),

strip.background = element_blank())+

facet_wrap(~Plot)+

scale_color_manual(values = c("Control" = "darkgrey", "Drought"="black")) +

xlim(0,0.0001)+

theme_cowplot()+

theme(legend.position = 'none')+

xlab(element_blank())

swtz.a_p2_CH4

swtz.a_p1_CH4 <- ggplot(swtz.a, aes(x= CH4, group=Treatment, colour=Drought)) +

#scale_y_continuous( labels=fancy_scientific)+

geom_density(adjust=1.5, alpha=.4,size=2)+

facet_grid(~Plot)+

theme(plot.title = element_blank(),

panel.grid.minor=element_blank(),

panel.grid.major=element_blank(),

axis.text.y = element_text(angle = 45, hjust = 1),

panel.grid.major.y=element_blank(),

#legend.position="none",

legend.title = element_blank(),

strip.background = element_blank())+

theme(legend.position = 'none')+

scale_color_manual(values = c("Control" = "darkgrey", "Drought"="black")) +

theme_cowplot()+

xlim(-450,650)+

xlab(element_blank())+

ylab(element_blank())+

geom_vline(xintercept=0, color="darkgrey", linetype="dashed")+

coord_flip()

swtz.a_p1_CH4

layout <- "

AAAA##

BBBBCC

BBBBCC

"

swtz.a_p2_CH4 + swtz.a_exp_CH4 + swtz.a_p1_CH4+plot_layout(design = layout,guides = 'collect')

##################################################################################################
