# Supplementary material for: Vegetation type, not the legacy of warming, modifies the response of microbial functional genes and greenhouse gas fluxes to drought in Oro-Arctic and alpine regions
Source: FEMS Microbiol Ecol. 2023 Nov 10;99(12):fiad145. doi: 10.1093/femsec/fiad145 (PMC10673709; doi:10.1093/femsec/fiad145)
Supplement: fiad145_Supplemental_Files [file fiad145_supplemental_files.zip › Supplementary data code 1 effect size graphs.docx]

Resistance.for.graphs <- read.delim("C:/Users/fryel/OneDrive - Edge Hill University/Resistance experiment 2/DNA stuff/qPCR/Resistance for graphs.txt")

View(Resistance.for.graphs)

attach(Resistance.for.graphs)

#

#Package preload

library(dotwhisker)

library(broom)

library(dplyr)

library(patchwork)

library(ggplot2)

### Note: Cohen's d effect sizes and confidence intervals have been calculated and compiled in an Excel file, which is read here.

Resistance.for.graphs$Species<- factor(Resistance.for.graphs$Species,levels = c("Cassiope", "Eriophorum","Ranunculus","Saxifraga"))

levels(Resistance.for.graphs$Species) <- list('Latnjajaure Wet meadow'="Ranunculus",'Latnjajaure Dry heath'="Cassiope", 'Latnjajaure Tussock tundra'="Eriophorum",'Val Bercla Alpine'="Saxifraga")

Resistance.for.graphs$Species

position_jitterdodge(

jitter.width = NULL,

jitter.height = 0,

dodge.width = 0.75,

seed = NA

)

pd <- position_dodge(0.5)

a <- ggplot(Resistance.for.graphs) +

theme_bw(base_size=13) +

aes(x = Gene, y = Resistance, linetype=Warming) +

geom_errorbar(aes(ymin = resistminCI, ymax = resistmaxCI), width = 0.5,position=pd) +

geom_point(size = 1.25,position=pd) +

geom_hline(yintercept = c(0,1,-1), linetype = "dashed", size = 0.15, colour = "slategrey") +

facet_wrap(~Species, nrow = 1) +

labs(x = "", y = "Standardised effect size: Resistance") +

theme(plot.title = element_text(vjust=0.5, face="bold"),

panel.grid.minor=element_blank(),

axis.text.x = element_text(angle = 45, hjust = 1),

panel.grid.major.y=element_blank(),

legend.position="bottom",

legend.title = element_blank(),

strip.background = element_blank(),

)+

ylim(c(-1.5, 1.5))# +

a # export at 1250 x 750

b <- ggplot(Resistance.for.graphs) +

theme_bw(base_size=13) +

aes(x = Gene, y = Resilience, linetype=Warming) +

geom_errorbar(aes(ymin = resilminCI, ymax = resilmaxCI), width = 0.5,position=pd) +

geom_point(size = 1.25,position=pd) +

geom_hline(yintercept = c(0,1,-1), linetype = "dashed", size = 0.15, colour = "slategrey") +

facet_wrap(~Species, nrow = 1) +

labs(x = "", y = "Standardised effect size: Resilience") +

theme(plot.title = element_text(vjust=0.5, face="bold"),

panel.grid.minor=element_blank(),

axis.text.x = element_text(angle = 45, hjust = 1),

panel.grid.major.y=element_blank(),

legend.position="bottom",

legend.title = element_blank(),

strip.background = element_blank(),

)+

ylim(c(-1.5, 1.5))# +

b # export at 1250 x 750

a/b + plot_layout(guides = 'collect') & theme(legend.position = 'bottom')
